# Cisplatin-induced increase in heregulin 1 and its attenuation by the monoclonal ErbB3 antibody seribantumab in bladder cancer

**DOI:** 10.1038/s41598-023-36774-1

**Published:** 2023-06-14

**Authors:** Thomas M. Steele, Maria Malvina Tsamouri, Salma Siddiqui, Christopher A. Lucchesi, Demitria Vasilatis, Benjamin A. Mooso, Blythe P. Durbin-Johnson, Ai-Hong Ma, Nazila Hejazi, Mamta Parikh, Maria Mudryj, Chong-xian Pan, Paramita M. Ghosh

**Affiliations:** 1 Research Service, VA Northern California Health Care System, Mather, CA USA; 2 Department of Urological Surgery, University of California Davis School of Medicine, 4860 Y Street, Suite 3500, Sacramento, CA 95817 USA; 3 Surgical and Radiological Sciences, School of Veterinary Medicine, University of California Davis, Davis, USA; 4 Division of Biostatistics, Department of Public Health Sciences, University of California Davis, Davis, CA USA; 5 Department of Biochemistry and Molecular Medicine, University of California Davis, Sacramento, CA USA; 6 Yosemite Pathology Medical Group, Inc., Modesto, CA USA; 7 Division of Hematology and Oncology, Department of Internal Medicine, University of California Davis, Sacramento, CA USA; 8 Department of Medical Microbiology and Immunology, University of California Davis, Davis, CA USA; 9 Brigham and Women’s Hospital, Harvard Medical School, Boston, MA USA

**Keywords:** Cancer therapy, Oncogenes, Urological cancer, Chemotherapy, Bladder

## Abstract

Cisplatin-based combination chemotherapy is the foundation for treatment of advanced bladder cancer (BlCa), but many patients develop chemoresistance mediated by increased Akt and ERK phosphorylation. However, the mechanism by which cisplatin induces this increase has not been elucidated. Among six patient-derived xenograft (PDX) models of BlCa, we observed that the cisplatin-resistant BL0269 express high epidermal growth factor receptor, ErbB2/HER2 and ErbB3/HER3. Cisplatin treatment transiently increased phospho-ErbB3 (Y1328), phospho-ERK (T202/Y204) and phospho-Akt (S473), and analysis of radical cystectomy tissues from patients with BlCa showed correlation between ErbB3 and ERK phosphorylation, likely due to the activation of ERK via the ErbB3 pathway. In vitro analysis revealed a role for the ErbB3 ligand heregulin1-β1 (HRG1/NRG1), which is higher in chemoresistant lines compared to cisplatin-sensitive cells. Additionally, cisplatin treatment, both in PDX and cell models, increased HRG1 levels. The monoclonal antibody seribantumab, that obstructs ErbB3 ligand-binding, suppressed HRG1-induced ErbB3, Akt and ERK phosphorylation. Seribantumab also prevented tumor growth in both the chemosensitive BL0440 and chemoresistant BL0269 models. Our data demonstrate that cisplatin-associated increases in Akt and ERK phosphorylation is mediated by an elevation in HRG1, suggesting that inhibition of ErbB3 phosphorylation may be a useful therapeutic strategy in BlCa with high phospho-ErbB3 and HRG1 levels.

## Introduction

Muscle invasive bladder cancer (MIBC) constitutes about 25% of all initial diagnoses of bladder cancer (BlCa) but 80% of deaths from this disease^1,2^. Recommended treatment for MIBC patients is neoadjuvant chemotherapy (NAC) using *cis*-diamminedichlorideplatinum (II) (cisplatin)-based chemotherapy (methotrexate, vinblastine, doxorubicin, and cisplatin (MVAC) or gemcitabine plus cisplatin (GC)) followed by radical cystectomy (RC)^3,4^. In practice, though, patients (who are overwhelmingly [67–75%] male^5^), often do not receive NAC^6,7^ and are considered for adjuvant chemotherapy (AC) with the same regimens. Recurrence occurs frequently and a number of assays have been introduced for early detection and monitoring of recurrence^8^. While AC increases time to recurrence, 50% of patients ultimately relapse^9^.

Recent developments indicate a number of additional treatments for patients who fail chemotherapy. Nivolumab, an immune-checkpoint inhibitor (ICI) targeting programmed death-1 (PD-1), is an established adjuvant treatment of MIBC^10^ that reaches an objective response rate (ORR) < 30%^11^, but ICIs are known to also cause autoimmune diseases^12^. Erdafitinib, a small molecule inhibitor of fibroblast growth factor receptor (FGFR), is active in platinum refractory BlCa expressing FGFR3 mutations and FGFR2/3 fusions^13^. Antibody drug conjugates (ADCs) enfortumab vedotin, a nectin-4-directed antibody and microtubule inhibitor conjugate^14,15^, and sacituzumab govitecan, an anti-Trop-2-antibody conjugated to a topoisomerase inhibitor^16^, also demonstrated activity in BlCa. RC48-ADC (disitamab vedotin), conjugating the anti-HER2 antibody hertuzumab to monomethyl auristatin E (MMAE)^17,18^, had activity in patients with HER2-amplified or -mutated BlCa that failed chemotherapy^19–22^. However, relatively few patients benefit from these treatments. Therefore, understanding the mechanism of cisplatin resistance and developing therapeutic strategies to overcome them are of utmost importance^23^.

One of the causes of cisplatin failure has been traced to cisplatin-induced activation of extracellular regulated kinase 1/2 (ERK1/2) (a mitogen activated protein kinase, MAPK)^24–26^, while phosphorylation of protein kinase B (PKB/Akt)^27,28^ was also increased by cisplatin treatment. The epidermal growth factor receptor (EGFR), and its effector Src, was shown to mediate cisplatin-induced upregulation of ERK phosphorylation. This caused acquired chemoresistance in non-small cell lung cancer (NSCLC) via phosphorylation of paxillin resulting in increased Bcl-2 expression and cell survival^26^, and increased Akt/mTOR phosphorylation via activation of p38MAPK^27–29^. However, this increase in Akt and ERK phosphorylation was also observed in systems where the EGFR/Src pathway was not active, indicating the presence of alternate mechanism by which the ERK and Akt pathways are activated by cisplatin, that has not previously been identified.

The EGFR family of receptor tyrosine kinases (RTK) (EGFR, ErbB2/HER2, ErbB3/HER3, ErbB4/HER4) plays a significant role in the growth and progression of MIBC^30,31^. RTKs are stimulated by ligands, including epidermal growth factor (EGF) for EGFR, and heregulins (HRG1/2) for ErbB3^32^, while HER2 is a constitutively-active orphan receptor^33^. Two other heregulins, HRG3-4, are ligands for ErbB4, which is not highly expressed in BlCa. HRG1-4 (not to be confused with *heme responsive genes*, *HRG*) are encoded by the genes *neuregulin* (*NRG1-4)*, also referred to as NEU. HRG1 has been known to be expressed in six different forms stemming from alternative splicing^34^^—^here we will use the most common form, heregulin1-β1 (type I). The RTKs of the EGFR family undergo homo- or heterodimerization following ligand binding, stimulating autophosphorylation, and signaling to downstream targets including the phosphatidylinositol 3-kinase (PI3K)/Akt and Ras/MAPK pathways^31^. ErbB3 contains six binding sites for the p85-regulatory subunit of PI3K, including Y1289^35^; while Y1328 mostly binds Shc, signaling to the MAPKs ERK1/2^36^. However, the trigger that activates these pathways downstream of cisplatin treatment has not been identified.

Although multiple ErbB inhibitors have been tested in clinical trials for BlCa^31^, trials of EGFR inhibitors were relatively unsuccessful. The EGFR inhibitor panitumumab failed to reach efficacy in combination with MVAC^37^, while a Phase III trial of the dual EGFR/HER2 inhibitor lapatinib following chemotherapy showed no significant benefit^38^. However, trastuzumab-deruxtecan (targeting HER2 and topoisomerase-I respectively) with nivolumab showed activity in HER2-overexpressing BlCa^39^, while disitamab-vedotin (which also targets HER2) was granted US-FDA breakthrough-therapy designation for patients with HER2-positive BlCa following platinum-based chemotherapy^19,40^. Significantly, disitamab-vedotin was also efficacious (ORR = 26.3%) in HER2-negative BlCa patients^21^. Since HER2 most commonly dimerizes with ErbB3 in BlCa^41^, these results may indicate parallel effects of ErbB3 in this disease. In support of this hypothesis, the pan-ErbB inhibitor Afatinib demonstrated significant activity in patients with platinum-refractory BlCa with *HER2* or *ERBB3* alterations^42^. These results indicate an increased role of ErbB3 in BlCa progression.

A number of specific ErbB3 inhibitors have been developed, including patritumab deruxtecan that reached Phase III in NSCLC; and seribantumab, lumretuzumab, elgemtumab, duligotuzumab, and istiratumab that reached Phase II^43^. The humanized monoclonal antibody, seribantumab (MM-121/SAR256212), that blocks the ligand binding domain of ErbB3, completed Phase II studies in breast^44–46^, ovarian^47,48^, pancreatic^49^, and NSCLC^50^, and was found to be effective in combination with trastuzumab^51^, and the EGFR inhibitors erlotinib^49^, gefitinib^52^, and cetuximab^53^. However, few studies investigated ErbB3 in cisplatin-resistant MIBC^54^.

Here, we demonstrate for the first time that cisplatin increases the levels of the ErbB3 ligand, HRG1, in BlCa cell lines and animal models, which in turn increases Akt and ERK phosphorylation. Inhibition of HRG1 binding to its receptor, and ErbB3 activation, with seribantumab, reduced tumor growth in immunocompromised mice bearing patient-derived xenograft (PDX) tumors that expressed high HRG1 levels and an activated HER2/ErbB3 axis. The current results indicate correlation between cisplatin-resistance and both ErbB3 expression and activation in the context of advanced BlCa.

## Results

### Cisplatin transiently increases ErbB3 phosphorylation at Y1328 and Akt phosphorylation at S473 in a PDX model of MIBC

**Figure 1:**
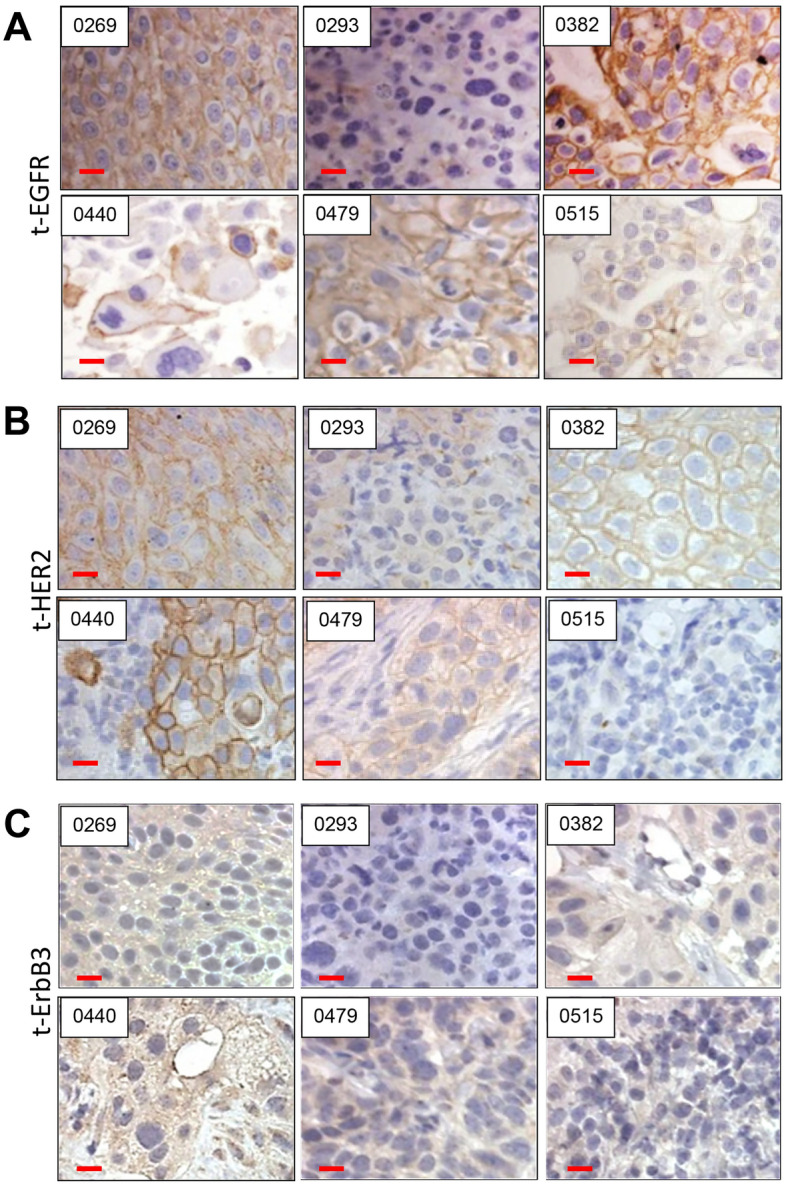
Expression of EGFR, HER2 and ErbB3 in bladder cancer patient derived xenografts. (**A**) Previously developed patient derived xenografts from bladder cancer tissue obtained from patients with urothelial carcinoma were implanted and cultured in NSG mice. The tumors were examined for EGFR expression. Immunohistochemistry in FFPE sections shows strong membrane staining in three samples (0269, 0382, 0479), and no or little staining in three (0293, 0440, 0515) (see also Supplementary Fig. 1A). **(B**) Investigation of HER2 levels showed strong membrane staining in one (0440), intermediate in three (0269, 0382 and 0479), and weak/absent in two (0293, 0515). (**C**) ErbB3 staining was more diffuse and was observed in all the tumors except 0293. Bar: 50 μm.

We previously reported the development of multiple PDX models of MIBC in immunocompromised mice^55^. The study identified the EGFR family as some of the most highly mutated/activated genes in MIBC. Hence, we compared the protein levels of this family in 6 PDX models that varied in response to cisplatin treatment (BL0269, BL0293, BL0382, BL0479, BL0515 and BL0440)^55^. IHC analysis demonstrated that EGFR expression was extremely high in BL0382, moderately high in BL0269 and BL0479, while levels in BL0293, BL0515 and in BL0440 were low (Fig. 1A). In contrast, HER2 was highly expressed in BL0440, moderately expressed in BL0269, BL0382 and BL0440, and weakly expressed in BL0293 and BL0515 (Fig. 1B). ErbB3 was also observed at moderately high levels in BL0269, BL0382, and BL0440 (Fig. 1C). These results supported previously identified gene expression of these RTKs in the PDXs by TCGA, IntOGen, and BGI Analyses reported elsewhere^55^ (Supplementary Fig. 1A). Although EGFR and HER2 were primarily localized in the membrane, ErbB3 was diffusely expressed in both the membrane and the cytoplasm, while ErbB4 was not detected.

**Figure 2:**
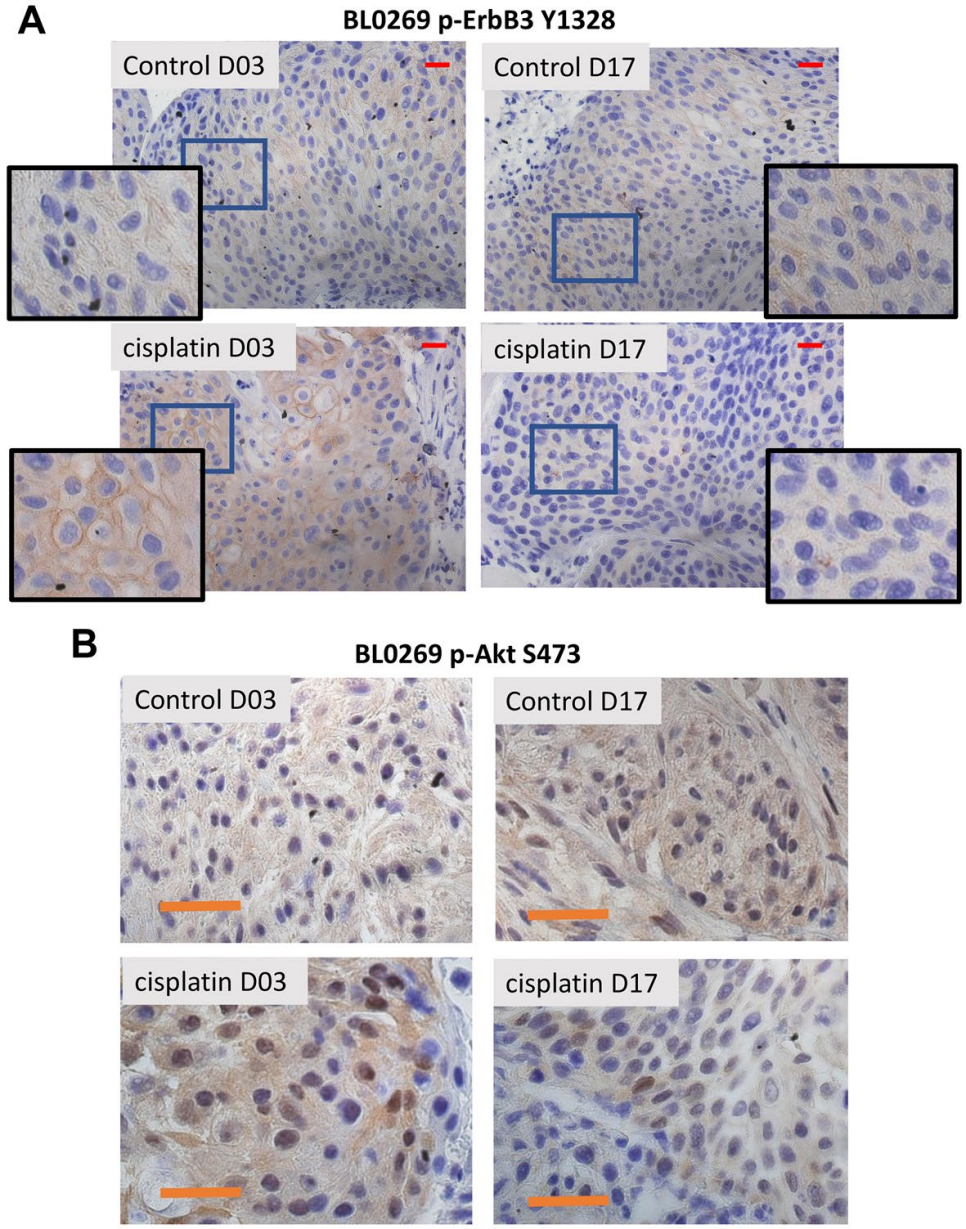
Effect of cisplatin on ErbB3 phosphorylation at Y1328 and Akt phosphorylation at S473 in the BL0269 PDX model. Mice bearing BL0269 PDX tumors (that express all three RTKs–EGFR, HER2, ErbB3), were treated with vehicle or 2 mg/kg cisplatin for 3 and 17 days; mice were euthanized, and tumors extracted, formalin fixed and paraffin-embedded. (**A**) Sections were cut and stained with antibodies to ErbB3 phosphorylated atY1328. (Scale Bar: 50 µm) Inset: Enlarged/magnified figures showing strong membrane staining of phospho-ErbB3(Y1328) in control treated cells but not in cisplatin-treated cells. Phospho-ErbB3 staining in control tumors were scored at + 1.5 whereas that in cisplatin-treated mice were scored at + 3 on day 3 but back to + 1 on Day 17. (**B**) Tumors were stained for phosphorylation levels of AKT at S473. (Scale Bar = 50 µm). Staining intensity was scored at + 1.5 in control tumors but increased to + 2 on day 3 and back to + 1 on day 17.

We investigated the effects of cisplatin on the EGFR family of RTKs in BL0269 since it expressed comparable levels of EGFR, HER2, and ErbB3 (Fig. 1). 6–8-week-old female NSG mice were implanted with BL0269 tumors and treated twice weekly with vehicle or 2 mg/Kg cisplatin as described in *Materials and Methods*. Tumors were collected after 3 and 17 days, which showed little alteration in ErbB3 expression upon cisplatin treatment (Supplementary Fig. 1B). The activity of the EGFR family of RTKs is typically measured by the phosphorylation levels of these kinases at tyrosine residues. Cisplatin-treated tumors showed no consistent difference in EGFR phosphorylation at Y845 and Y1045 for both time points (Supplementary Fig. 2A, B), while HER2 phosphorylation at Y1248 increased sporadically after 17 days (Supplementary Fig. 2C). On the other hand, ErbB3 phosphorylation at Y1328 increased after 3 days but decreased after 17 days compared to control (Fig. 2A). ErbB3 is a strong activator of the PI3K pathway and contains 6 binding sites in its kinase domain for the PI3K subunit p85^35^, which is known to signal downstream to Akt. Hence, we investigated the phosphorylation of Akt in the same BL0269 model. Cisplatin increased Akt phosphorylation at S473 at day 3 but decreased it by day 17 (Fig. 2B). Similar increases in ERK phosphorylation has also been reported^24–26^. The transient increase in both ErbB3 phosphorylation and in Akt phosphorylation suggest the existence of a pathway that may be of importance in the regulated transmission of signals to downstream targets.

### Cisplatin increases ERK and Akt phosphorylation in bladder cancer cells independent of chemosensitivity

**Figure 3:**
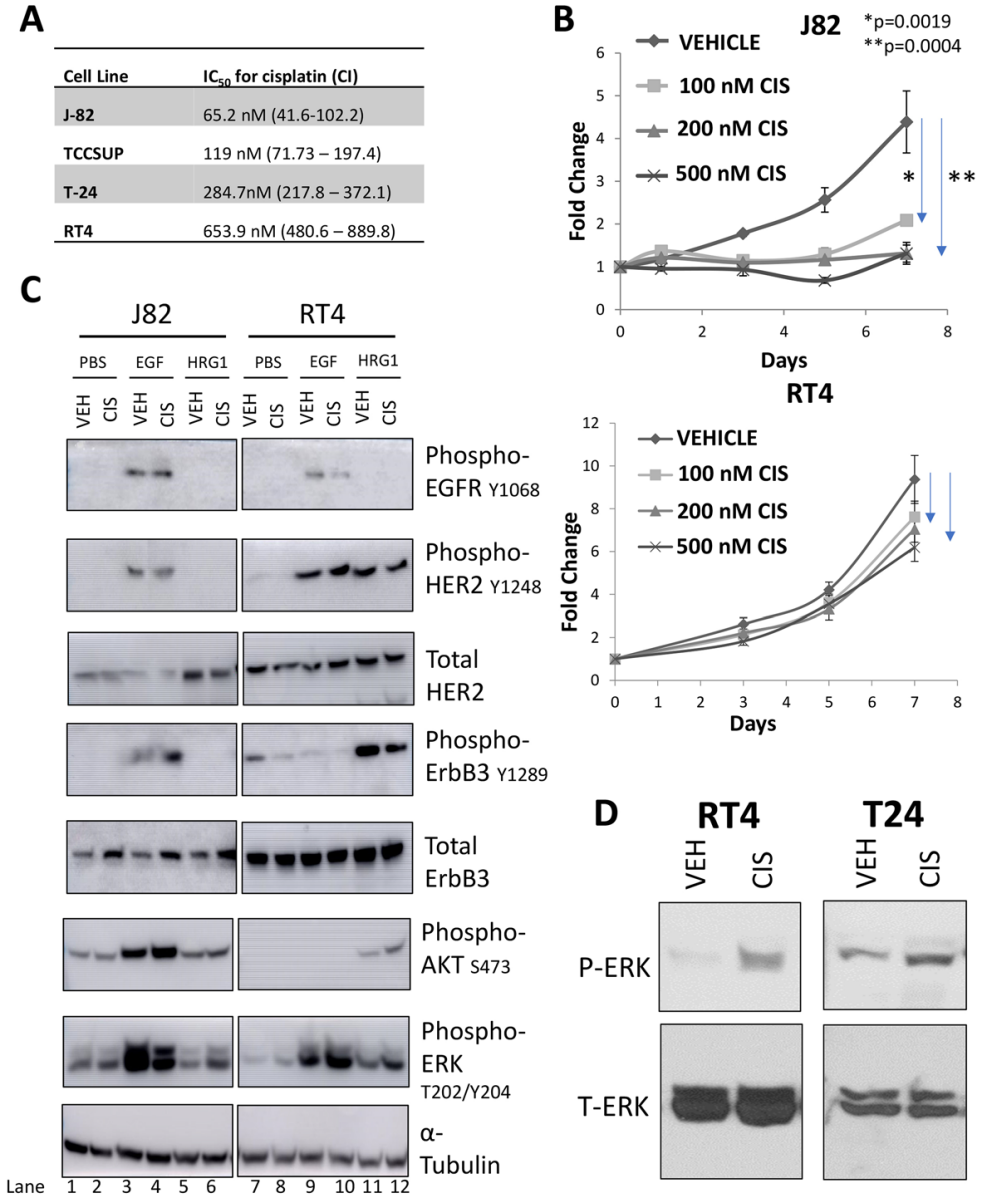
Effect of cisplatin on chemosensitive J82 and chemoresistant RT4 cells stimulated with PBS (control), EGF to stimulate EGFR or HRG1 to stimulate ErbB3. (**A**) Comparison of the effects of cisplatin in various bladder cancer cell lines. Cell lines are organized in order of their resistance to cisplatin. IC_50_ values are presented together with the respective confidence intervals (CI). (**B**) MTT assay of J82 (upper) and RT4 cells (lower) treated with varying concentrations of cisplatin and collected over 7 days. These data are representative of the ones used to calculate the IC_50_ values. (**C**) Immunoblot of J82 and RT4 cells treated with vehicle or 200 nM cisplatin for 72 h, then stimulated with vehicle, 10 ng/mL of EGF, or 50 ng/mL of HRG for 15 min prior to collection. Lysates were run on a gel, transferred to a membrane and the latter subjected to Western blotting using antibodies to various proteins as indicated. Bands were quantitated and normalized to the corresponding bands in the α-tubulin lanes (marked from 1 to 12 respectively). (**D**) Immunoblots comparing the effects of 72 h of 200 nM cisplatin on ERK phosphorylation in cisplatin resistant T24 and RT4 bladder cancer cells. Note that in both cases, ERK phosphorylation is increased with cisplatin.

We next investigated whether the changes in ErbB3 phosphorylation by cisplatin are ligand-mediated. There have been multiple reports describing cisplatin efficacy in various BlCa lines^56–58^. Based on these reports, we tested four BlCa cell lines of varying cisplatin sensitivity (2 from male patients and 2 from female patients) with the goal to analyze cisplatin’s effects on ErbB3 signaling. First, we determined cisplatin’s effect on viability of J82, TCCSUP, T24, and RT4 cell by MTT assay. IC_50_ values showed that J82 cells are the most sensitive (IC_50_ 65.2 nM) and RT4 cells are the least sensitive (IC_50_ 653.9) (Fig. 3A, B). Immunoblot comparison of protein expression in the four lines showed that RT4 cells expressed much higher levels of ErbB3 compared to the rest (Supplementary Fig. 3A). RT4 cells also expressed higher levels of HER2, but EGFR levels were comparable among all the lines (not shown).

To test the effects of cisplatin on the phosphorylation of the EGFR family of RTKs and their downstream targets, the most and least cisplatin-sensitive cell lines, J82 and RT4, were treated with 200 nM cisplatin or vehicle for 72 h followed by a 15-min stimulation with the EGFR ligand EGF or the ErbB3 ligand HRG1 (Fig. 3C). In J82 cells, EGFR(Y1068), HER2(Y1248), or ErbB3(Y1289) phosphorylation was observed only upon EGF treatment, and of these, only ErbB3(Y1289) phosphorylation increased with cisplatin treatment (Fig. 3C, lanes 3, 4). In contrast, in RT4 cells, EGF stimulated EGFR phosphorylation, and HRG1 stimulated ErbB3 phosphorylation, while HER2 phosphorylation was stimulated in both conditions. In these cells, cisplatin reduced EGFR phosphorylation at Y1068 (Fig. 3C, lanes 9, 10) and ErbB3 phosphorylation at Y1289 (Fig. 3C, lanes 11, 12), but it did not significantly affect HER2 phosphorylation at Y1248. Thus, J82 cells, similar to the chemosensitive PDX model BL0269, demonstrated an increase in ErbB3 phosphorylation upon treatment with cisplatin.

In J82 cells, both AKT and p42/44MAPK (ERK1/2) were constitutively phosphorylated but were further stimulated only by EGF (Fig. 3C, lanes 3, 4). In contrast, in RT4 cells, Akt phosphorylation was stimulated only by HRG1 (Fig. 3C, lanes 11,12) while ERK phosphorylation was stimulated by both EGF and HRG1 (with a larger response from EGF) (Fig. 3C, lanes 9–12). Additionally, in RT4 cells, cisplatin further increased Akt and ERK phosphorylation, especially in the presence of HRG1 (Fig. 3C, lanes 9–12). These studies indicate an intricate relationship between HRG1 stimulation and cisplatin treatment. To determine whether similar effects were seen in other cisplatin-resistant lines we tested the effect of cisplatin in the highly-invasive and high-grade T24 cells^59^. Similar to RT4, cisplatin induced a sharp increase in ERK phosphorylation in T24 cells, indicating the ubiquitous nature of this phenomenon in bladder cancer cells (Fig. 3D).

### Correlation between ERK and ErbB3 phosphorylation in patients with Urothelial Carcinoma (UC)

We investigated whether ERK phosphorylation correlates with ErbB3 phosphorylation in 55 patients with BlCa who underwent radical cystectomy at the University of California Davis Comprehensive Cancer Center (UCDCCC) (Supplementary Table 1). The patient cohort had a median age of 68 (spanning 36 to 85 years of age), and the majority (81.8%) were male. Most of the patients were Caucasians (61.8%) with the rest identifying as African American (1.8%), Asian (1.8%), other (5.5%), or undisclosed (29.1%); only 7.55% were listed as Hispanic or Latino. 25.5% underwent chemotherapy and 29.1% had passed away at the time of reporting.

**Figure 4:**
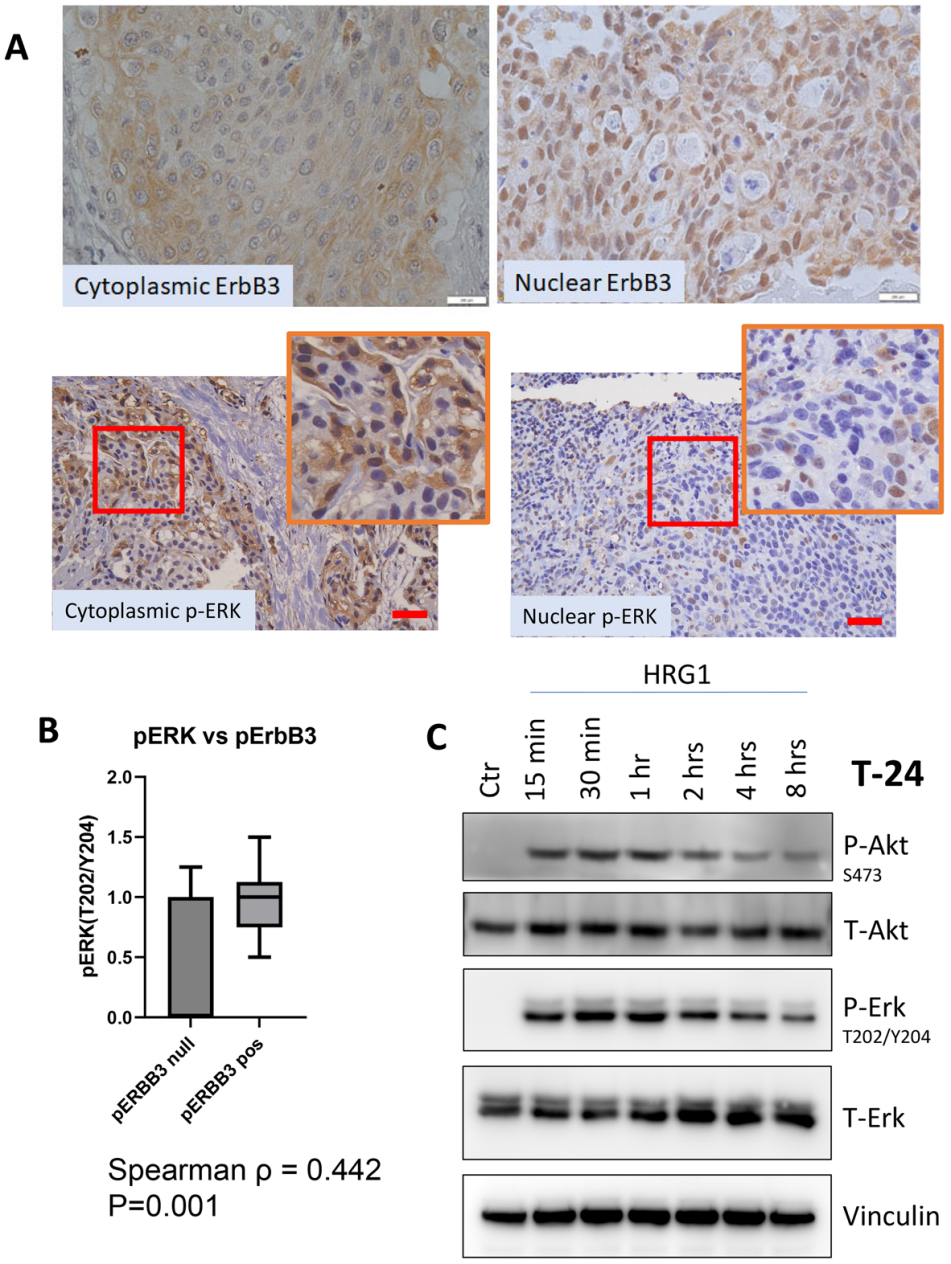
ERK1/2 phosphorylation at Thr 202/Tyr 204 in tumor sections from patients with urothelial carcinoma (UC). (**A**) Tissue microarrays (TMA) containing bladder cancer cores from 55 patients in triplicate were sectioned and stained for phospho-ERK1/2 (T202/Y204). Of the 55 patients, 16 (29%) did not express phospho-ERK (upper left); 5 (9%) expressed phospho-ERK in the nucleus only (upper right), in 10 (18.18%), phospho-ERK was cytoplasmic only (lower left), while in the remaining 24 (43.6%), it was expressed in both the nucleus and the cytoplasm (lower right). Brown staining indicates phospho-ERK. Blue indicates hematoxylin counterstain. Bar: 15 µM. Inset shows enlarged pictures from the same. (**B**) Correlation of ERK phosphorylation with ErbB3 phosphorylation in corresponding patients. ERK phosphorylation was scored separately in the nucleus and the cytoplasm. Spearman ρ = 0.44 denote moderate correlation, which was nevertheless significant. (**C**) Immunoblot demonstrating effect of HRG1 on Akt and ERK phosphorylation. Cisplatin resistant T-24 cells were stimulated with 50 ng/ml HRG1 for the indicated lengths of time. Lysates were run on a gel and blotted for phospho-Akt (Ser 473) and phospho-ERK (Thr 202/Tyr 204). Note that short term treatment with HRG1 induces an acute increase in Akt and ERK phosphorylation but tapers off after prolonged exposure.

Tissue microarrays (TMA) containing three representative cores from each patient were stained for total ErbB3, phospho-ErbB3, or phospho-ERK as described in *Materials and Methods*. ErbB3 expression was detected in both the nucleus and in the cytoplasmic/membranous region (Fig. 4A). None of the markers correlated with age, gender, race, or ethnicity. Of the 55 patients, only 39 (70.9%) expressed phospho-ERK, and of the latter 10/39 (25.64%) expressed phospho-ERK exclusively in the cytoplasm, while 5/39 (12.82%) expressed phospho-ERK exclusively in the nucleus; the remaining 2439 patients (61.54%) expressed it in both compartments (Fig. 4A). ERK phosphorylation was higher in tissues from patients who experienced early death compared to those who did not (Supplementary Fig. 3B). The phosphorylation of ErbB3 at Y1289 significantly correlated with the phosphorylation of ERK at T202/Y204 (Spearman ρ = 0.44, *p* = 0.001) (Fig. 4B).

Finally, we investigated whether the effect of cisplatin on ERK and Akt phosphorylation may be attributed to upstream activation of ErbB3. Since ErbB3 is phosphorylated by HRG1, we stimulated T-24 cells with 50 ng/ml HRG1 and followed its effects over time. HRG1 initially caused an increase in Akt phosphorylation at Ser 473 and ERK1/2 phosphorylation at T202/Y204, followed by an eventual decrease, likely due to receptor desensitization following chronic ligand exposure^60^ (Fig. 4C). This response was analogous to cisplatin’s effect on ErbB3 phosphorylation over a prolonged period.

### Cisplatin increases short term ErbB3 phosphorylation via upregulation of HRG1

**Figure 5:**
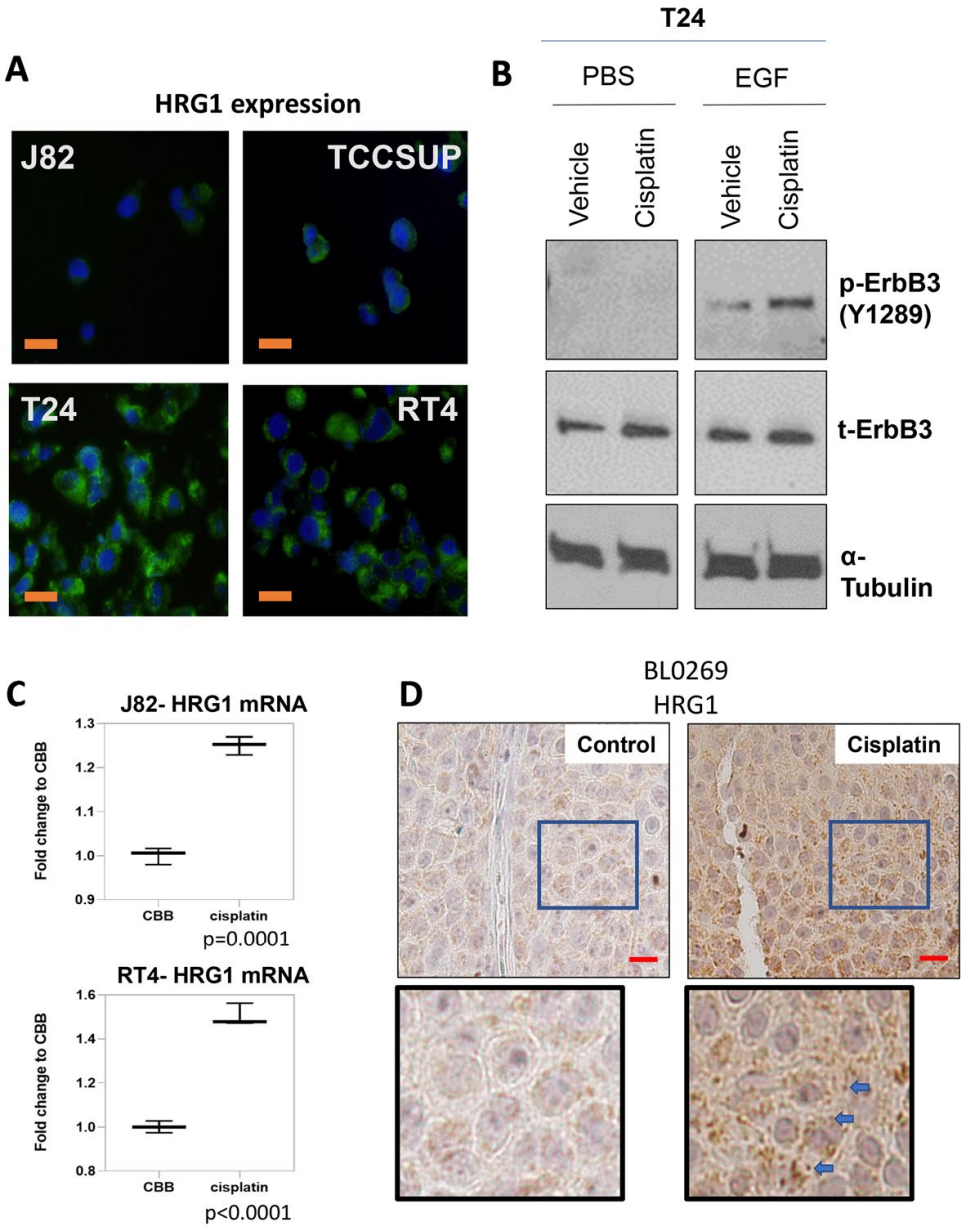
Cisplatin increases ErbB3 signaling via upregulation of HRG1. (**A**) Comparison of HRG1 expression in four BlCa cell lines—cisplatin sensitive J82 and TCCSUP and cisplatin resistant T24 and RT4. To eliminate differences in cell adhesion all cells were pelleted, and cell pellets were formalin-fixed and paraffin-embedded, then sections cut and stained by immunofluorescent techniques using an anti-HRG1 antibody and FITC-labelled secondary antibody. The slides were counterstained with DAPI to stain for nuclear material. Note that T24 and RT4 cells express significantly higher levels of HRG1 compared to J82 and TCCSUP. (**B**) Immunoblot of T24 treated with vehicle or 200 nM cisplatin for 72 h and stimulated for 15 min before collection with 10 ng/ml EGF or control. (**C**) HRG1 mRNA levels of cisplatin sensitive J82 and cisplatin resistant RT4 cells treated with vehicle or 200 nM cisplatin for 72 h. Details of primer used for the analysis is provided in *Materials and Methods*. (**D**) Immunohistochemistry of HRG1 in the BL0269 model when treated with vehicle or cisplatin (17 days). Mice bearing BL0269 PDX tumors were treated with 2 mg/kg cisplatin for 17 days**.** Sections from the tumors were stained with anti-HRG1 antibody. (Scale Bar = 25 µm).

Since HRG1 is a significant inducer of ErbB3 phosphorylation and downstream ERK phosphorylation and cisplatin had a significant effect on ERK phosphorylation, we investigated if cisplatin altered HRG1 levels. First, we investigated whether the expression of HRG1 correlated with the response of BlCa cells to cisplatin. Unsurprisingly, immunofluorescence studies confirm that HRG1 levels were significantly higher in cisplatin-resistant T24 and RT4 cells compared to cisplatin-sensitive J82 and TCCSUP cells (Fig. 5A). We validated these results by immunocytochemistry (Supplementary Fig. 4). The highly-invasive and high-grade T24 cells^59^ expressed the highest level of HRG1, and hence, we investigated whether cisplatin affected ErbB3 phosphorylation levels in these cells (Fig. 5B). Similar to J-82 cells, T-24 also demonstrated an increase in ErbB3 phosphorylation at Y1289 upon treatment with cisplatin.

Next, we explored whether cisplatin affected the levels of the ErbB3 ligand HRG1 in J82 and RT4 cells. 200 nM cisplatin treatment for 72 h increased HRG1 mRNA levels in both cell lines. In J82 cells, HRG1 mRNA increased by only about 25% after cisplatin treatment, whereas cisplatin increased HRG1 mRNA levels by 50% in RT4 (Fig. 5C). Finally, we examined whether these increases in the ErbB3 ligand are also observed in vivo. Examination of BL0269 tumors revealed a significant increase in HRG1 protein levels following cisplatin treatment compared to control (Fig. 5D). HRG1 localized predominantly in the cytoplasm and along the plasma membrane as punctate dots, and the intensity of the dots increased significantly following cisplatin treatment. These results indicate not only that cisplatin-resistant models exhibit high intrinsic levels of HRG1, but also that these levels increase further upon cisplatin treatment.

### Seribantumab reduces ErbB3 Phosphorylation and tumor growth in cisplatin-resistant BlCa cell line and PDX models

**Figure 6:**
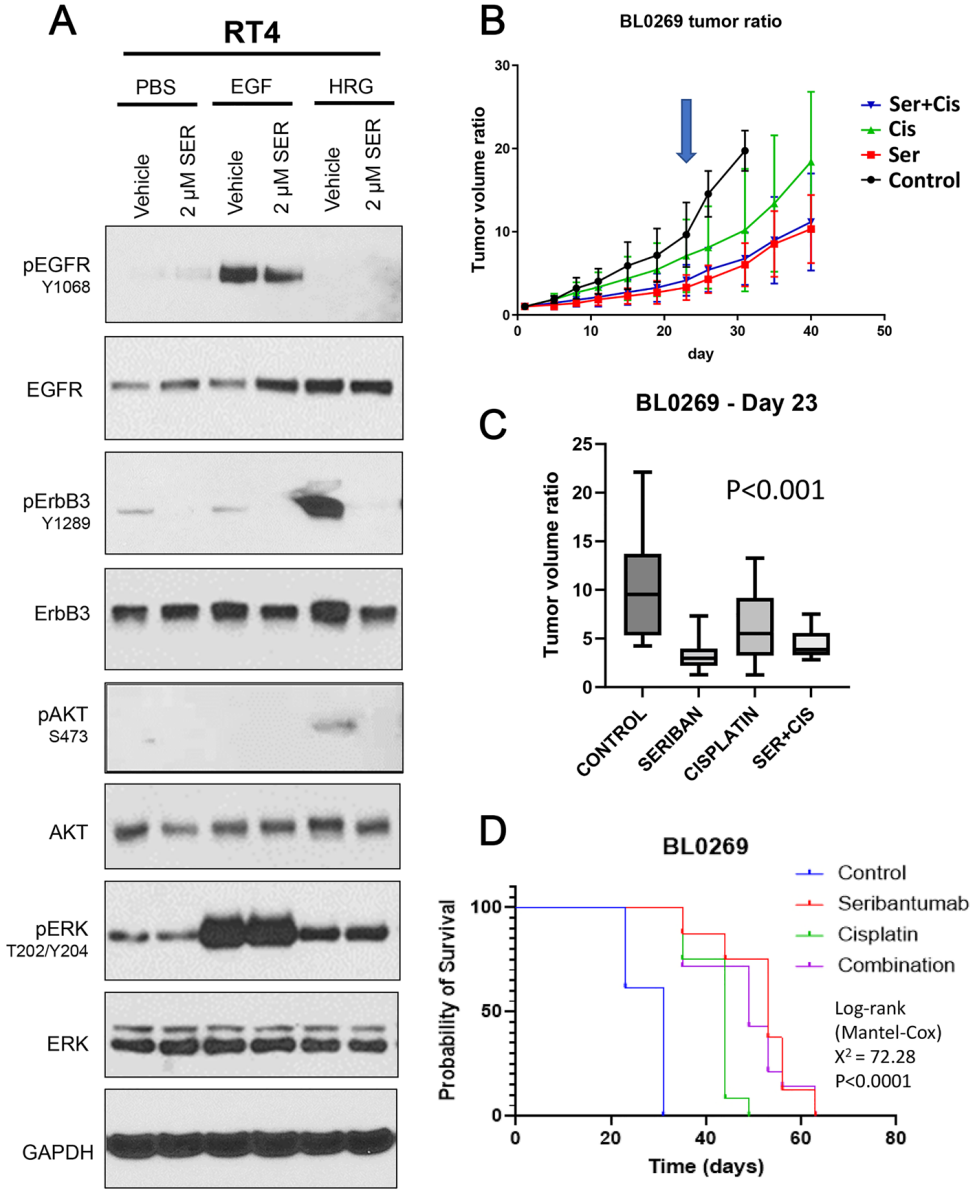
Seribantumab inhibits ErbB3 phosphorylation and signaling and reduces tumor growth in cisplatin resistant BlCa. (**A**) Immunoblot of RT4 cells treated with vehicle or 2 µM seribantumab for 72 h then stimulated with vehicle, 10 ng/ml EGF, or 50 ng/ml HRG for 15 min prior to collection. (**B**) Tumor volume progression over time, in mice implanted with BL0269 PDX tumors, showing greater effect of seribantumab compared to cisplatin. Mice were treated with 2 mg/Kg cisplatin, 10 mg/Kg seribantumab, the combination, or placebo (n = 7 per group) twice weekly for three weeks and then monitored for up to 40 days. Blue arrow indicates the three week point after which treatment was stopped. (**C**) Box and whiskers plot showing median tumor volume between the four groups on day 23, after which treatment was stopped. (**D**) Corresponding survival curve of BL0269 PDX showing effectiveness of single agent seribantumab over cisplatin and similar efficacy as the combination.

To investigate if we could disrupt the connection between cisplatin-induced increase in HRG1 and subsequent activation of Akt and ERK downstream, we sought a method to suppress or prevent HRG1-mediated ErbB3 activity. Studies have shown that seribantumab, a monoclonal antibody targeting the ligand binding domain of ErbB3, inhibits ErbB3 signaling and tumor growth when a HRG1 autocrine loop is present^52,61^. Hence, we tested the effects of seribantumab in cisplatin-resistant RT4 cells. 2 µM seribantumab treatment for 72 h greatly reduced HRG1-stimulated ErbB3 phosphorylation at Y1289 but still reduced it under all other conditions (Fig. 6A). It also decreased EGF-stimulated EGFR phosphorylation at Y1068, which likely indicates inactivation of EGFR/ErbB3 dimers. Seribantumab also reduced HRG1-mediated Akt and ERK phosphorylation whereas EGF-stimulated ERK activity was not affected, indicating that seribantumab’s effect was specific to HRG1/ErbB3 binding and not EGF/EGFR binding.

Since cisplatin increases HRG1 levels and subsequently leads to increased Akt and ERK phosphorylation, we investigated whether seribantumab prevent these effects**.** Therefore, we examined the overall effect of seribantumab as a single agent and in combination with cisplatin in the chemoresistant PDX line BL0269. We implanted immunocompromised mice with BL0269 PDX tumors and treated them twice weekly with vehicle, 2 mg/kg cisplatin, 10 mg/kg of seribantumab, or the combination (Fig. 6B). Cisplatin treatment alone (n = 7) reduced tumor growth slightly compared to vehicle (n = 7) by day 23 (*p* = 0.0429) (Fig. 6C), consistent with the small effect of cisplatin previously reported in this model^55^. In contrast, single agent seribantumab treatment significantly reduced tumor growth in the BL0269 PDX (n = 9, *p* = 0.0006) (Fig. 6B,C). Time to euthanasia was determined by tumor volume and/or mouse health condition, and mice treated with seribantumab alone survived longer compared to those with cisplatin or vehicle (Fig. 6D). Log-rank (Mantel Cox) test for the comparison of survival groups show that survival of control mice was significantly lower than that of the treated groups (Supplementary Fig. 5A). However, the combination treatment did not have any significant effects over that of seribantumab alone in the BL0269 model, and ErbB3 expression in the combination was similar to that from seribantumab alone (Supplementary Fig. 5B). However, seribantumab significantly reduced ErbB3 phosphorylation at Y1289 (Supplementary Fig. 5C). Our results suggest that the high intrinsic HRG1 levels of these tumors (Fig. 5D), enable efficacy of seribantumab; further elevation of HRG1 levels do not further increase its actions. Overall, the effect of seribantumab in the chemoresistant invasive model of BlCa exceeded that of cisplatin and could be effective as a single agent in cisplatin-resistant urothelial carcinoma.

### Single-agent seribantumab is effective in a PDX model of BlCa that overexpresses HER2

**Figure 7:**
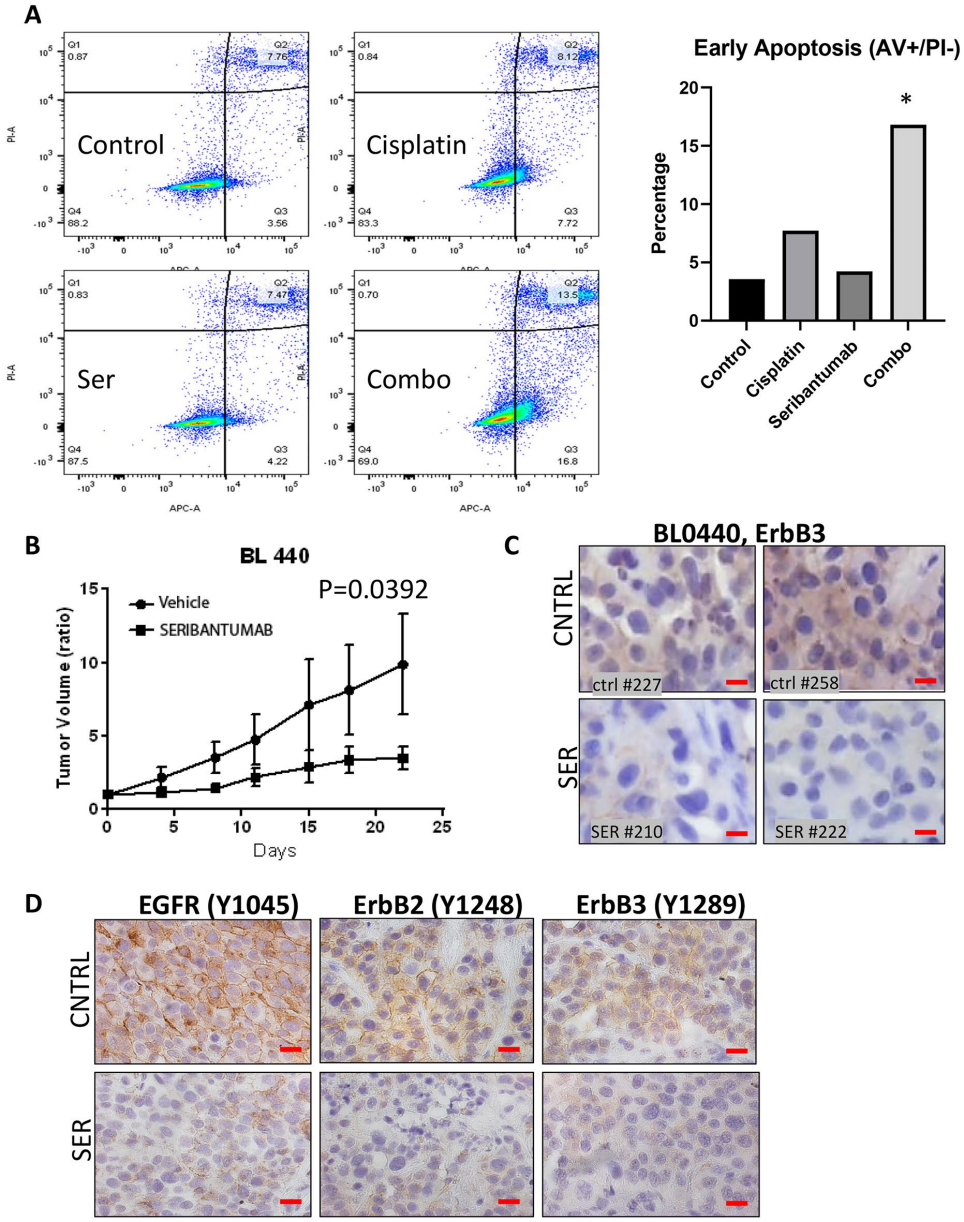
Seribantumab reduces tumor growth in a cisplatin–sensitive PDX model. (**A**) J82 cells were treated with vehicle, 200 nM cisplatin, 2 µM seribantumab or the combination for 72 h, after which cells were trypsinized, and unfixed cells were stained with propidium iodide (PI) and Annexin V (AV). Stained cells were then assayed by flow cytometry as described^62^. Data obtained was analyzed to obtain fractions that represent live cells (unstained), early apoptosis (Annexin V stained), late apoptosis (both AV and PI treated) and in necrosis (PI only). (**B**) Tumor volume progression over time and ErbB3 staining, in mice dually implanted with BL0440 PDX tumors. Mice were treated with 10 mg/kg seribantumab or placebo twice weekly for three weeks and then monitored for up to 10 days. (**C**) Immnohistochemistry of ErbB3 staining in the BL0440 model when treated with control or cisplatin after 35 days. (Scale Bar = 25 µm). (**D**) Representative images of immunohistochemical analysis for EGFR, HER2 and ErbB3 phosphorylation in BL0440 PDXs showing that seribantumab treatment for 21 days suppressed all ErbB receptor phosphorylation.

If seribantumab is effective as a single agent, then it could potentially have efficacy in cisplatin-sensitive BlCa with active ErbB3 signaling as well. To test this hypothesis, we investigated the effect of the ErbB3 inhibitor in J82 cells that experience an increase in ErbB3 upon cisplatin treatment (Supplementary Fig. 3A). We had observed that cisplatin increases the level of HRG1 in J82 (Fig. 5C), hence we postulated that seribantumab may attenuate the effects of increased HRG1 and increase responsiveness to cisplatin, preventing acquired resistance to this agent. J82 cells were treated with vehicle, 200 nM cisplatin, 2 µM seribantumab, or the combination for 72 h. Cells were processed for flow cytometry following Annexin V and Propidium Iodide to identify cells undergoing apoptosis or necrosis respectively as described elsewhere^62^. We noted that in these cisplatin sensitive tumors, the apoptotic fraction increased > twofold with cisplatin alone, but seribantumab alone had no effect (Fig. 7A). However, the combination of seribantumab with cisplatin increased apoptosis by 4.7-fold, and the fraction of cells undergoing necrotic cell death 1.7-fold (Supplementary Fig. 6A). Thus, despite high HER2 levels, in these cells, seribantumab as a monotherapy was ineffective due to low HRG1 levels. However, it could affect the cells only where HRG1 had been increased to a certain level by cisplatin.

These results suggested that seribantumab may be effective in any tumor that express high levels of HRG1 and/or ErbB3 phosphorylation. To determine whether these effects of seribantumab affected tumor growth, we tested the effect of seribantumab as a single-agent in the cisplatin-responsive BL0440 PDX model^55^, which expresses high levels of HER2 and ErbB3 (Fig. 1). We treated BL0440-bearing mice with 10 mg/kg of seribantumab twice a week for three weeks by i.p. for up to 35 days (Fig. 7B). After 25 days, the control tumors had increased in volume by 9.88-fold, while tumors in the seribantumab-treated mice had only increased by 3.5-fold (*p* = 0.0024) (Supplementary Fig. 6B). Single-agent treatment with seribantumab was more effective than single-agent weekly doses of 6 mg/kg trastuzumab (HER2 monoclonal antibody), indicating the importance of ErbB3 in HER2 positive tumors (Supplementary Fig. 6B). ErbB3 levels were suppressed by seribantumab in BL0440 tumors (Fig. 7C), whereas trastuzumab did not alter ErbB3 levels (Supplementary Fig. 6C). Single agent seribantumab also suppressed EGFR phosphorylation at Y1045, HER2 phosphorylation at Y1248, and ErbB3 phosphorylation at Y1289 (Fig. 7D). Thus, inhibition of ErbB3 signaling had a profound effect on growth of this HER2 overexpressing tumor.

## Discussion

In this paper, we investigate the mechanism by which cisplatin causes an increase in Akt and ERK phosphorylation in BlCa cells, which has previously been related to chemoresistance. Collectively, our data indicate that cisplatin treatment alters ErbB3 signaling in BlCa by affecting levels of the ErbB3 ligand HRG1. Comparison of BlCa cells and tumor lines with varying sensitivity to cisplatin demonstrated that cisplatin induced a transient increase in ErbB3 phosphorylation at Y1328, ERK1/2 phosphorylation at T202/Y204, and Akt phosphorylation at S473. Investigation of the mechanism of the transient increase in ErbB3 phosphorylation revealed that treatment with cisplatin leads to an increase in HRG1 mRNA and subsequent protein levels, which stimulates ErbB3 phosphorylation and leads to an increase in Akt and ERK activation, followed by eventual receptor desensitization. To prevent the transient increase, we treated BlCa cell lines and PDX models with seribantumab, which targets ErbB3 ligand binding. Seribantumab prevented HRG1-induced ERK and AKT phosphorylation in vitro and significantly reduced tumor growth in both cisplatin-sensitive and -resistant BlCa PDX models. Our results demonstrate that inhibition of the HER2/ErbB3 axis is effective in BlCa exhibiting high HRG1-induced ErbB3 phosphorylation.

Cisplatin is the foundation of first-line combination chemotherapy used in standard-of-care treatment for MIBC in both NAC and AC for primary BlCa, as well as in treatment-naïve metastatic BlCa^4^. However, patients are often refractory or develop resistance to this treatment^63^, with mechanisms of chemoresistance ranging from loss of long non-coding RNAs^64^ to disruptions in the DNA damage repair pathways^23^. Disruptions in the signal transduction pathways emanating from RTKs are also major causes of chemoresistance; hence, newer therapies including HER2-targeting ADCs such as RC48 have been developed for this population. Surprisingly, RC48-ADC was also efficacious (objective response rate (ORR) = 26.3%) in HER2-negative BlCa patients^21^. Thus, it is worthwhile to consider other closely related targets in BlCa, like ErbB3. Here, we show for the first time that cisplatin upregulates HRG1 in BlCa. These findings support previous studies indicating correlation between cisplatin resistance and NRG1/HRG1^65,66^, labelled an oncogene that stimulates the dimerization of ErbB3 with HER2 or EGFR^67^.

Receptor desensitization following prolonged ligand exposure, despite initial activation, typically is followed by autophosphorylation at sites promoting endosomal encapsulation and eventual degradation^68^. In the case of ErbB3, phosphorylation at Y1289, the p85^PI3K^-binding site^33^, and at Y1328, the Shc binding site^36^, responded to the increase in HRG1 by cisplatin. Activation of the receptor is complete when it binds to its dimerization partners EGFR and HER2 (as most BlCa do not express ErbB4^55^). Remarkably, in RT4 cells, HRG1 stimulated HER2 and ErbB3 phosphorylation (indicating the formation of HER2/ErbB3 dimers), and not EGFR phosphorylation. On the other hand, EGF stimulated phosphorylation in EGFR and HER2, but not ErbB3 (suggesting the formation of EGFR/HER2 heterodimers and EGFR homodimers). These RTKs then activated their downstream targets, Akt and ERK. Our data demonstrate that in human BlCa tissue, ERK phosphorylation is correlated with ErbB3 phosphorylation, suggesting the significance of the Y1328 site, and PDX models also demonstrated correlation with Akt phosphorylation. These results explain how an increase in HRG1 by cisplatin may upregulate ERK and Akt phosphorylation.

HRG1 is transcribed by the gene *neuregulin 1 (NRG1)* and our data indicate that cisplatin increases *NRG1* transcription rates. It is important to note that cisplatin crosslinks with purine bases on DNA, interfering with DNA repair mechanisms, thereby inducing apoptosis^69^. Several transcription factors have been associated with cisplatin resistance, including ATF2, E2F1, NR4A and Sp1^70^. Some of these transcription factors, including ATF2 and Sp1, are also transcriptional regulators of *NRG1*. While outside of the scope of the current article, it may be speculated that cisplatin resistance may activate these transcription factors resulting in *NRG1* transcription.

Seribantumab, which prevents HRG1 binding, reduced tumor growth in both cisplatin-sensitive BL0440 and cisplatin-resistant BL0269 PDX models. Our data demonstrate significant HRG1 expression in these tumors, which was further increased by cisplatin treatment. These results suggest that seribantumab was able to suppress the growth of the tumor by inhibiting the intrinsic expression of HRG1 in them, whereas cisplatin was ineffective due to high HRG1 levels. In contrast, J82 cells express low levels of HRG1—therefore, seribantumab was ineffective in these cells as a monotherapy. However, as demonstrated, cisplatin treatment increases HRG1 levels thereby allowing seribantumab efficacy. Therefore, in these cells, the combination of seribantumab and cisplatin was highly effective.

Note that BL0440, although cisplatin sensitive, was gemcitabine resistant^55^. Additionally, BL0440 had high HER2 and ErbB3 levels but low EGFR levels, suggesting that ErbB signaling may take place through HER2/ErbB3 dimers. Our data shows that under conditions of high HRG1 and active ErbB3, ErbB3 inhibition reduces BlCa tumor growth. In high-HER2-expressing BL0440 tumors and high-EGFR-expressing BL0269, inhibition of HRG1-ErbB3 binding reduced tumor volume. Despite cisplatin increasing HRG1 levels, combining cisplatin and seribantumab had no benefit above seribantumab monotherapy in a chemoresistant PDX model. Development of ADCs which conjugate ErbB3 antibodies with payload chemotherapeutic agents may have utility in urothelial carcinoma, especially in patients who are refractory to platinum-based therapy.

**Figure 8:**
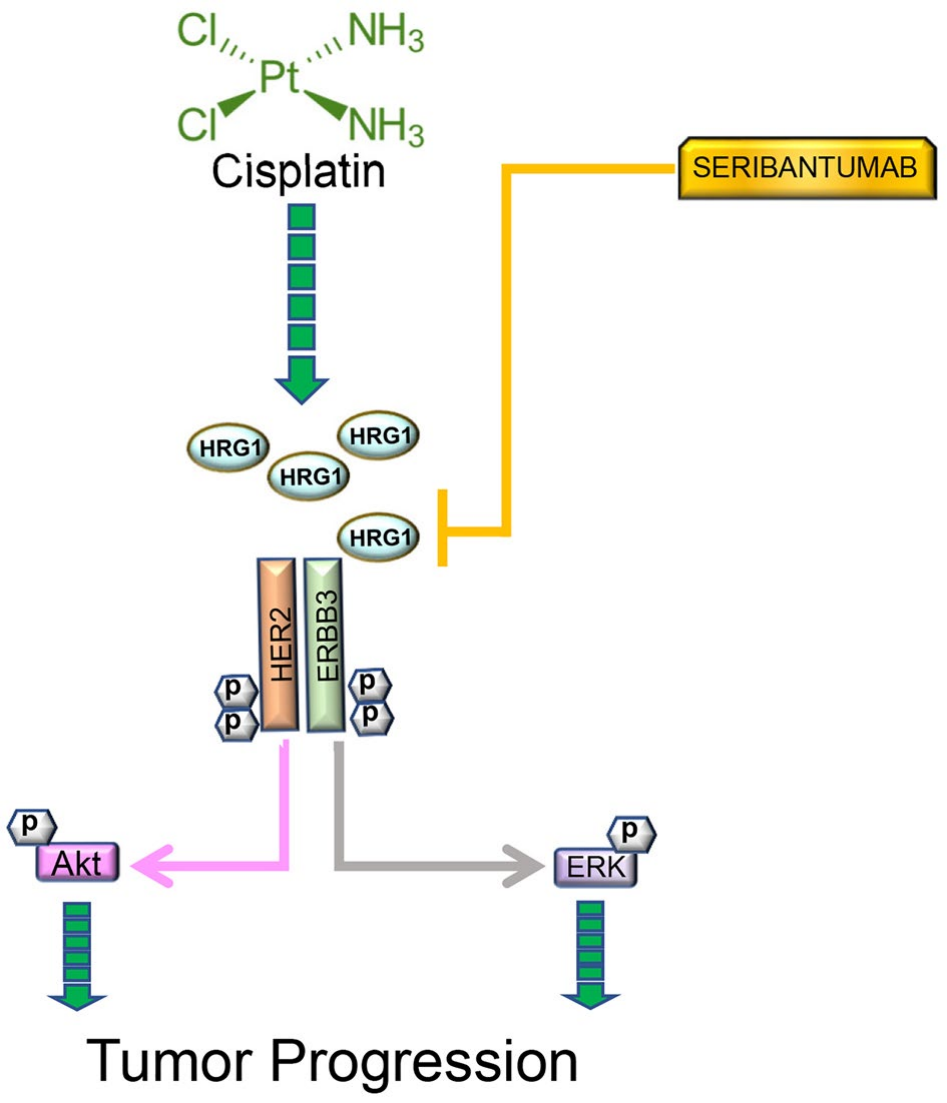
Schematic describing ErbB3 signaling affected by cisplatin.

In conclusion, HER2/ErbB3 signaling may be impactful in cisplatin-resistant BlCa, but both cisplatin-sensitive and -resistant BlCa positive for HRG1 may be susceptible to ErbB3 inhibitors like seribantumab if they express high HRG1 levels. Overall, our data suggest that ErbB3 inhibition may be a viable treatment option for patients with MIBC that express HRG1 and rely on HRG1/ErbB3 signaling whether cisplatin-sensitive or -resistant (Fig. 8). To our knowledge, this is the first attempt to correlate cisplatin-resistance with ErbB3 expression and activation in the context of advanced BlCa and may affect patient selection for upcoming drugs targeting the HER2/ErbB3 axis.

## Methods

### Urothelial carcinoma (UC) tissue microarrays

All human tissues were collected with approval from the Institutional Review Board (IRB), UC Davis (protocol #293,828) and all experiments were performed in accordance with University of California Davis (UCD) IRB guidelines and regulations. All patients provided informed consent to participate under the IRB-approved protocol. Tissue microarrays (TMA) representing a total of 55 BlCa patients in quadruple were obtained in a deidentified manner from the UCD Comprehensive Cancer Center (UCDCCC) biorepository that collected the tissues. Annotated data from the 55 patients are presented in Supplementary Table 1. Immunohistochemistry (IHC) was performed as previously described^71,72^ using a monoclonal anti-ErbB3 antibody, anti-HRG1 antibody and anti-phospho-Erk1/2 antibody (Supplementary Table 2). Marker levels were assessed separately in the cell membrane/ cytoplasm and in the nucleus and the scoring system was as follows: 0: no staining (< 10%) 1: weak (10–40%), 2: medium (40–70%), 3: strong (70–100%). IHC figures were obtained directly from the camera attached to the microscope and incorporated in MS Powerpoint.

### Cell lines

The cell lines *T24*, *TCCSUP*, *RT4*, and *J-82* were purchased from American Type Culture Collection (ATCC), Manassas, VA (Supplementary Table 3). Cells were maintained in RPMI 1640 medium (Invitrogen, Grand Island, NY) supplemented with 10% FBS (Gemini Bio Products, West Sacramento, CA) and 1% Pen/Strep (Invitrogen, Grand Island, NY) unless otherwise indicated and grown in 5% CO_2_ at 37 °C. Cells were routinely tested for mycoplasma.

### Pharmacologic treatments and MTT and apoptosis assays

Seribantumab was kindly provided by Merrimack Pharmaceuticals, Inc. (Cambridge, MA), while cisplatin was purchased from Fisher Scientific (Waltham, MA) and Selleck Chem (Houston, TX). Seribantumab was pre-prepared in a proprietary solution from Merrimack Pharmaceuticals (corresponding vehicle also provided) and cisplatin was dissolved in platinum binding buffer (CBB) (3 mM NaCl and 1 mM sodium phosphate). Epidermal Growth Factor (EGF; 10 ng/mL) and Heregulin-beta1β-1 (HRG1; 50 ng/mL) was purchased from Peprotech and used to stimulate cells 15 min before collection or across a time-course. Cell viability in the presence of inhibitors was estimated by 3-[4,5-Dimethylthiazol-2yl]-2,5-diphenyl-tetrazolium bromide (MTT) assay as described before^71,72^. Apoptosis was estimated by flow cytometry in propidium iodide and Annexin V-stained unfixed cells as described by us^62^.

### Immunoblotting, immunofluorescence, immunohistochemistry, qPCR

Western blotting^71,72^. Immunohistochemistry^62,73^ and Immunofluorescence^62^ was performed as previously described. The antibodies used in this study can be found in Supplementary Table 2. qPCR primers were designed using Primer-Blast from NCBI. HRG1 Forward: 5’-ATC AGT ATC CAC AGA AGG AGC A-3’ and Reverse-5’-TGG CAG CGA TCA CCA GTA AA-3’. All western blots were retrieved as digital images and the pictures were cropped using MS powerpoint to fit into the figures. Uncropped blots with band quantification are presented in the Supplementary Material. Band intensity was normalized to that of a loading control and represented as fold changes over control lanes. Wherever possible, bands were used from the same membrane, however, in many cases, the same membrane could not be used for phospho- total- protein bands of the same target. This is because they run at exactly the same spot, thereby confusing the results. In these cases, we ran two parallel gels from the same lysates, loaded in the same identical order, using the same amount of lysate (in ug) on to each apparatus that is run in parallel in the same apparatus, and using the same power supply. If in doubt, at the end of the experiment, we use Ponceau Red staining to ensure that the same amount of protein had been loaded in each gel. Usually, this ensures that the two gels get treated equally.

### Patient derived xenograft studies

Clinical information and cancer specimens from BlCa patients for PDX development was collected under the University of California Davis IRB Protocol No. 218204. Animal studies were approved by the Institutional Animal Care and Use Committee (IACUC), UC Davis and all experiments were performed in accordance with UC Davis IACUC #17794. Experimental descriptions in this manuscript complies with the ARRIVE guidelines. PDX models (BL0269 (JAX# TM00015), BL0293 (JAX# TM00016), BL0382 (JAX# TM00020), BL0440 (JAX# TM00024), BL0479 (JAX# TM00026), BL0515 (JAX# TM00031)) were implanted subcutaneously into the flanks of 6–8 week old female NOD/scid/gamma (NSG) mice^55^. When tumors reached 150–200 mm^3^, mice were randomly assigned (no *apriori* set criteria were used for the assignment) and treated twice weekly—vehicle (30% PEG-300, 1% Tween80, 1% DMSO), 2 mg/Kg cisplatin, 10 mg/Kg seribantumab by intra-peritoneal (ip) injection and the combination of cisplatin and seribantumab (n = 7 per group, exceptions noted in Results or in Figure Legends). Isoflurane (1–5%) by inhalation was used for anesthesia, if needed. For combination treatments, seribantumab was delivered first followed by cisplatin. All animals were included in the final calculations. Treatments continued for 3 weeks after which the mice were observed for a total of 40 days. Tumor volume (lengthXwidth^2^X0.5) and animal body weight were recorded twice weekly, in no particular order. Mice were euthanized by pentobarbital overdose followed by cervical dislocation when at least one of the following humane endpoints were reached: (i) tumor volume of 1.5 cm^3^, (ii) 15 mm tumor length towards any direction, (iii) tumor ulceration, (iv) more than 20% reduction in animal body weight. Tumors were collected and formalin fixed, and paraffin embedded, then sectioned and stained with antibodies as described elsewhere^62,73^. The sections were read by a blinded Veterinary Pathologist and scored as described^62,73^.

### Statistical analysis

IC_50_ was calculated from MTT results performed in triplicate at multiple doses of drug using GraphPad Prism software (v9.0.0). Normal distribution was assessed using Shapiro–Wilk test and data were analyzed accordingly. Significance between two sets of data in mouse tumors or in vitro assays were calculated by paired t-test using at least three independent values. For in vivo experiments, a sample size of at least n = 6 was chosen assuming α = 0.05, β = 0.2, to achieve 80% power. FlowJo software was used to analyzed flow cytometry experiments and GraphPad Prism software (v9.0.0) was used for the rest of the analyses in animal and cell line studies. *P* value < 0.05 was considered statistically significant. Western blots were scanned by Image J software and band intensities were reported as intensity of the immunoblot developed using the specific antibody normalized to that of the corresponding band in the loading control (e.g. GAPDH).

TMA IHC data were summarized across positions by taking the median of non-missing values. The correlation between age and marker expression was calculated using Spearman (nonparametric) correlations. Marker expression was compared between levels of categorical variables with two levels using Wilcoxon rank-sum tests. Marker expression was compared among levels of categorical variables with more than two levels using Kruskal–Wallis tests (nonparametric ANOVA), followed by Dunn tests in the case of a significant global test. Analyses were conducted using R version 4.0.2 (2020–06-22), with Dunn tests conducted using the R package FSA, version 0.8.30.

## Supplementary Information

Supplementary Information.

Supplementary Figures.

## Data Availability

All data generated or analyzed during this study are included in this published article [and its supplementary information files].
