# Supplementary material for: Cisplatin-induced increase in heregulin 1 and its attenuation by the monoclonal ErbB3 antibody seribantumab in bladder cancer
Source: Sci Rep. 2023 Jun 14;13:9617. doi: 10.1038/s41598-023-36774-1 (PMC10267166; doi:10.1038/s41598-023-36774-1)

SUPPLEMENTARY TABLES

Supplementary Table 1: Subject Characteristics

Age at Path Date	(n=55)
Mean (SD)	66.3 (11.7)
Median (Range)	68 (36--85)
Gender	
F	10 (18.2%)
M	45 (81.8%)
Race	
African American	1 (1.8%)
Asian	1 (1.8%)
Caucasian	34 (61.8%)
Other	3 (5.5%)
Not Listed	16 (29.1%)
Ethnicity	
Hispanic or Latino	4 (7.55%)
Not Hispanic or Latino	34 (54.55%)
Other	8 (15.09%)
Not Listed	7 (13.21%)
NEC	
Chemotherapy	14 (25.5%)
No Chemo reported	41 (74.5%)
Deceased	
Not Deceased	39 (70.9%)
Subject Deceased	16 (29.1%)

Supplementary Table 2: Antibodies used in this study

Antigen	Source	Catalog No	Dilution	Application
Actin	Santa Cruz Biotechnology	sc-1616	**"1:1000"**	WB
AKT	Santa Cruz Biotechnology	#4685	**"1:1000"**	WB
phospho-AKT(S473)	Cell Signaling Technology	#9271	**"1:500"**	WB
phospho-AKT(S473)	Cell Signaling Technology	#3787	**"1:50"**	IHC
Cleaved Caspase- 3	Cell Signaling Technology	#9661	**"1:1000"**	WB
Cleaved- PARP (D214)	Cell Signaling Technology	#9546	**"1:1000"**	WB
EGFR	Cell Signaling Technology	#2232	**"1:500"**	WB
EGFR	Invitrogen	#280005	**"1:40"**	IHC
phospho-EGFR (Y1045)	Cell Signaling Technology	#2237	**"1:50"**	IHC
phospho-EGFR (Y1068)	Cell Signaling Technology	#2234	**"1:500"**	WB
phospho-EGFR (Y1068)	Cell Signaling Technology	#3777	**"1:500"**	WB
phospho-EGFR (Y845)	Cell Signaling Technology	#2231	**"1:50"**	IHC
ErbB2/HER2	Santa Cruz Biotechnology	sc-284	**"1:500"**	WB
ErbB2/HER2	DAKO	#A0485	**"1:200"**	IHC
phospho-ErbB2 (Y1248)	Millipore	#06-229	**"1:100"**	IHC
phospho-ErbB2 (Y877)	Cell Signaling Technology	#2241	**"1:250"**	WB
phospho-ErbB2(Y1248)	Cell Signaling Technology	#2247	**"1:250"**	WB
ErbB3	Cell Signaling Technology	#12708	**"1:500"**	WB
ErbB3	Santa Cruz Biotechnology	sc-285	**"1:500"**	WB
ErbB3	Cell Signaling Technology	#12708	**"1:250"**	IHC
phospho-ErbB3 (Y1289)	Cell Signaling Technology	#4791	**"1:500"**	WB
phospho-ErbB3 (Y1289)	Cell Signaling Technology	#4791	**"1:100"**	IHC

phospho-ErbB3 (Y1328)	Cell Signaling Technology	#8017	**"1:250"**	IHC
ErbB4	Santa Cruz Biotechnology	sc-283	**"1:500"**	WB
ERK	Santa Cruz Biotechnology	sc-93	**"1:1000"**	WB
phospho-ERK1/2 (T202/Y204)	Cell Signaling Technology	#9107	**"1:500"**	WB
phospho-ERK1/2 (T202/Y204)	Cell Signaling Technology	#9101	**"1:50"**	IHC
GAPDH	Millipore	MAB374	**"1:1000"**	WB
NRG1/HRG1	ThermoFisher	MA5-12896	**"1:100"**	IHC
PARP	Cell Signaling Technology	#9532	**"1:1000"**	WB
α-tubulin	Cell Signaling Technology	#2125	**"1:1000"**	WB

Supplementary Table 3: Characteristics of cell lines used in this study

Cell line	Patient Gender	Patient Age	Disease
T24	Female, Caucasian	82 years	Transitional Cell Carcinoma, infiltrating
RT4	Male, Caucasian	63 years	Transitional cell Papilloma, superficial
J-82	Male, Caucasian	58 years	Transitional Cell Carcinoma, infiltrating
TCCSUP	Female	67 years	Transitional Cell Carcinoma - grade IV

SUPPLEMENTARY FIGURES AND LEGENDS

Supplementary Figure 1. (A) Expression of EGFR family of RTKs and their ligands in various PDX tumors. We previously reported the expression of these genes (Pan CX, et al. PLoS One. 2015 Aug 13;10(8):e0134346). **(B) BL0269 tumors collected after 3- and 17-days following cisplatin treatment showed little alteration in HER2 or ErbB3 expression.** Mice bearing BL0269 tumors were treated with vehicle or cisplatin and sacrificed after 3 or 17 days. Tumors were extracted, formalin fixed, and paraffin embedded. Then the FFPE samples sectioned and stained for HER2 and ErbB3 as indicated (bar: 50 μm).

A

Gene_Id	BL0269	BL0293	BL0382	BL0429	BL0440	BL0479	BL0515	
EGFR	21.0464	18.1222	774.834	13.4476	10.6429	58.0531	5.29813	
ERBB2	51.2582	19.2033	59.1518	134.383	158.051	42.2478	2.72069	
ERBB3	46.4868	0.439931	55.5352	98.1242	46.0066	16.3607	5.14123	
ERBB4	0.016168	0.00443	0.077381	0.202483	0.076507	0.012297	0	
EGF	0.019461	0.004914	2.79154	0.113574	0.005308	0.087321	0.006496	EGFR ligands
TGFA	8.46481	0.772886	9.36473	22.4229	26.4098	13.1027	4.29631	
HBEGF	8.81754	38.1933	3.07479	9.55732	6.64756	23.7702	10.701	
BTC	3.66545	1.09723	5.61878	6.82015	2.73333	1.55123	0.150942	
EREG	0.936432	9.26914	5.81843	6.61556	10.5626	8.00116	2.20566	
AREG	1.38585	9.11948	4.45696	2.38864	9.79421	23.0393	7.93021	
NRG1	4.65335	0.046631	0.046317	0.196113	0.343242	26.1146	3.95403	ErbB3 ligands
NRG2	0.017822	4.40665	0.017461	0.139362	0.055458	0.053952	1.50369	
NRG3	0	0	0	0.462223	0	0	0	ErbB4 ligands
NRG4	1.80216	1.01439	5.42594	3.02877	0.690328	0.375798	0.876469	

B

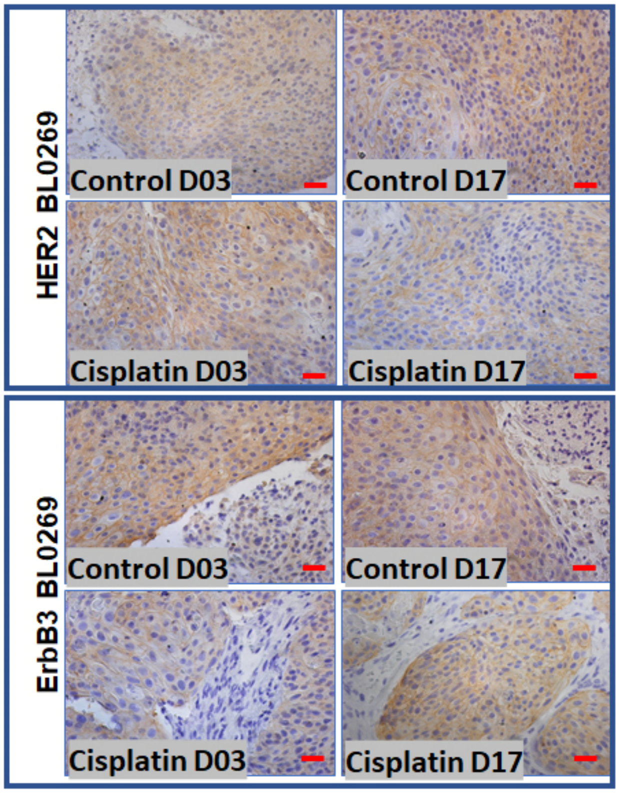

Supplementary Figure 2. BL0269 tumors collected after 3- and 17-days following cisplatin treatment showed little alteration in EGFR phosphorylation.
Mice bearing BL0269 tumors were treated with vehicle or cisplatin and sacrificed after 3 or 17 days. Tumors were extracted, formalin fixed, and paraffin embedded. Then the FFPE samples sectioned and stained for various phospho-proteins as indicated (bar: 50 μm).

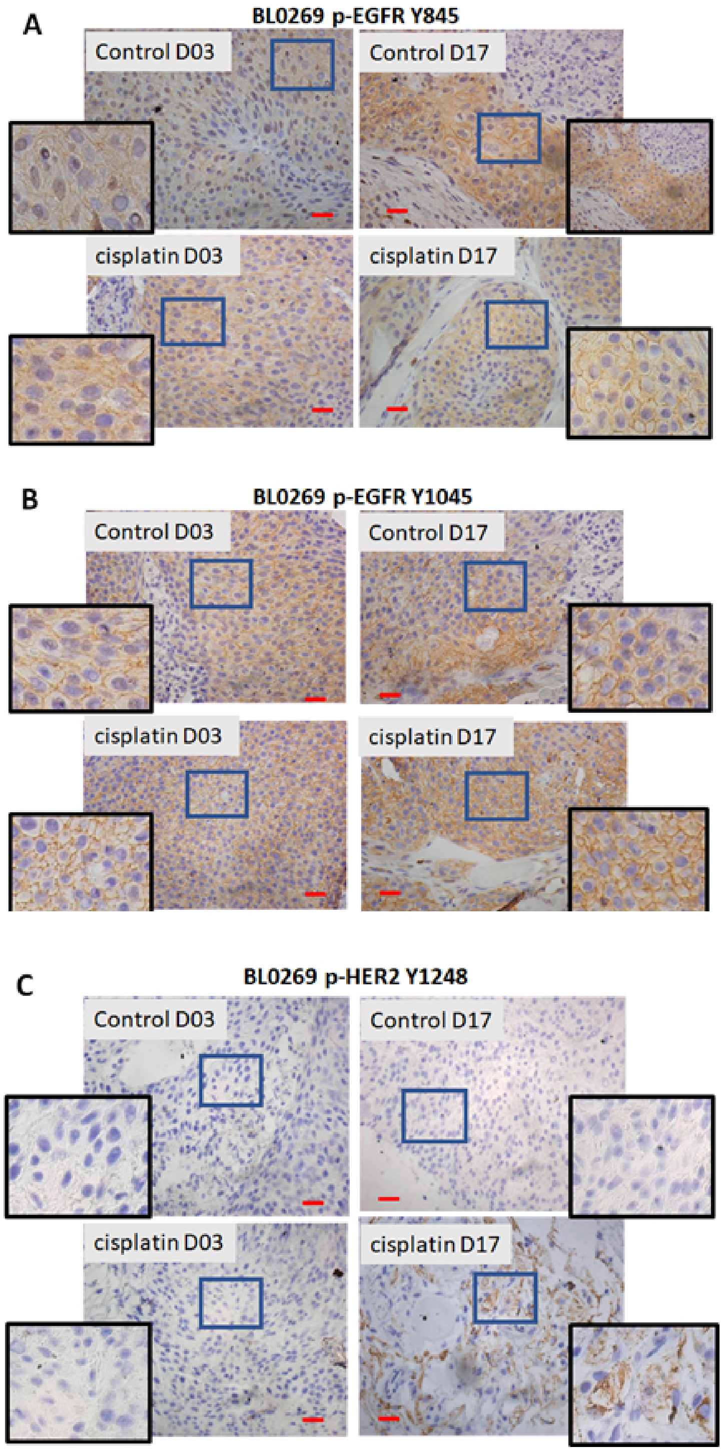

Supplementary Figure 3: Effects of cisplatin treatment of cisplatin on bladder cancer cell lines. **A).** T24, RT4, TCCSUP and J82 cells were treated with platinum binding buffer (3 mM NaCl and 1 mM sodium phosphate) (CBB) or 200 nM cisplatin in CBB for 72 hours. Immunoblots were subjected to anti-phospho-ErbB3 (Y1289) antibody or total ErbB3. Note that ErbB3 levels were much higher in RT4 cells compared to the rest, and therefore, we are showing here both an exposure that shows the expression of ErbB3 in T24 and J82 cells (TCCSUP did not appear to express detectable levels of ErbB3) and another exposure that showed the relative levels of ErbB3 in RT4 cells. **B).** Correlation of ERK phosphorylation with patient death, which was determined at the time of analysis. ERK phosphorylation was scored separately in the nucleus and the cytoplasm. Patient death correlated with nuclear ERK phosphorylation but not phosphorylation of cytoplasmic ERK1/2, indicating that it is the genomic effects of ERK phosphorylation that regulate patient survival.

A

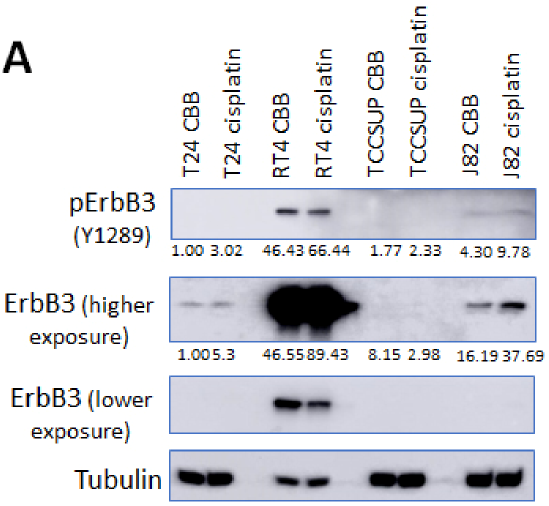

B Marker Expression by Death

	Not Deceased	Subject Deceased
PErk.N		
N	39	16
Median (Range)	0 (0--1)	0.5 (0--1)
PErk.C		
N	39	16
Median (Range)	0.5 (0--1.5)	0.5 (0--1.2)

Supplementary Figure 4. Increased expression of HRG1 in cisplatin resistant T24 and RT4 cells compared to cisplatin sensitive J82 and TCCSUP.

(A) The four cell lines were pelleted and the cell pellets formalin fixed and paraffin embedded. The FFPE cell pellets were then sectioned and stained for HRG1. Results indicate that T24 and RT4 cells express higher HRG1 (brown staining, see inset) compared to J82 and TCCSUP (bar: 50 uM).

(B) Extrinsic HRG1 localizes to the cell membrane. Cells were cultured in medium containing charcoal stripped serum to remove all steroids and growth factors including HRG1. Then the cells were treated for 72 hours with 200 nM cisplatin, which increased intrinsic HRG1 (localized to the cytosol). However, at the end of that period, 50 ng/ml extrinsic HRG1 was added to the medium for 15 minutes, then the cells were washed and fixed. Fixed cells were stained for HRG1. The extrinsic HRG1 localized to the cell membrane (bar: 15 μM).

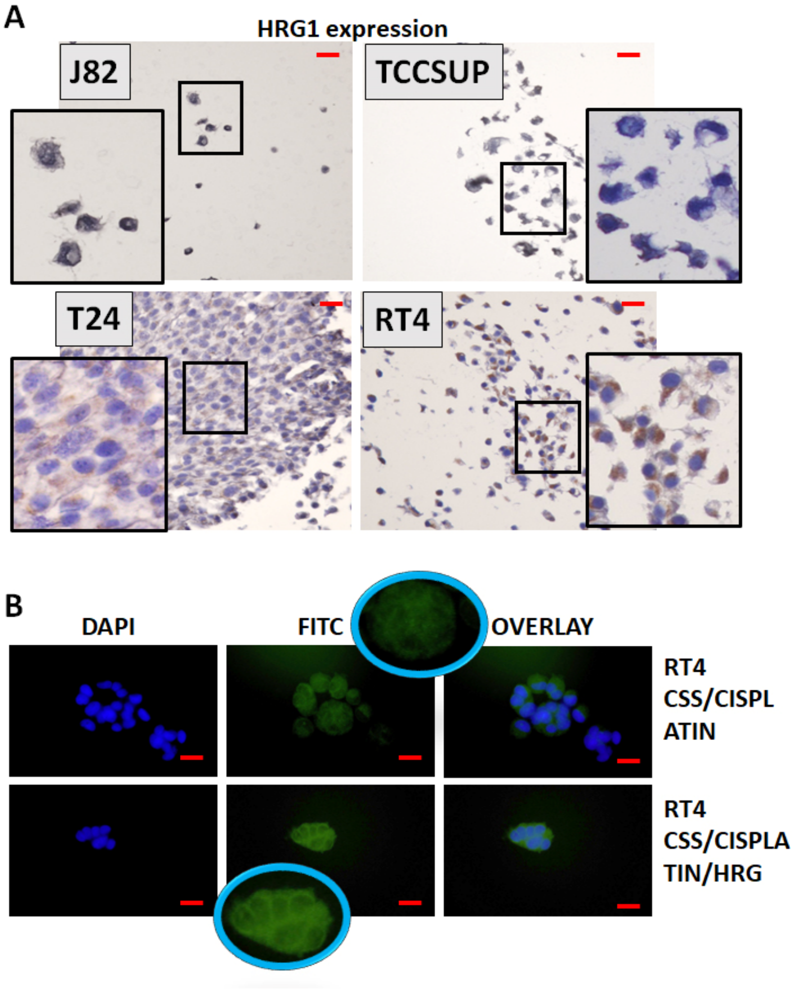

Supplementary Figure 5. Effect of the combination of cisplatin and seribantumab/MM-121 on ErbB3 protein levels in BL0269 tumors.

A) Log-rank (Mantel Cox) test for the comparison of survival groups. **B)** Immunocompromised mice bearing BL0269 tumors treated with vehicle (control), 2 mg/Kg cisplatin, 10 mg/Kg seribantumab (MM-121) and the combination of the two were formalin fixed and paraffin embedded. Sections were stained for ErbB3 levels by immunohistochemistry- (bar: 25 µm). **C).** Representative tumors from the four groups that were obtained following euthanasia of the mice at the end of the experiment. The tumors were formalin fixed and paraffin embedded, after which the blocks were sectioned and stained for ErbB3 phosphorylation (Y1289) (bar: 50 µm). Inset: Enlarged figures showing ErbB3 phosphorylation in the four groups.

A Log-rank Mantel-Cox test for Comparison of Survival Groups

	All groups	Cont. vs Ser.	Cont. vs. Cis.	Cont. vs Comb.
Chi square	72.28	28.51	23.01	25.78
df	3	1	1	1
P value	<0.0001	<0.0001	<0.0001	<0.0001
P value summary	****	****	****	****
Are the survival curves significantly different?	Yes	Yes	Yes	Yes

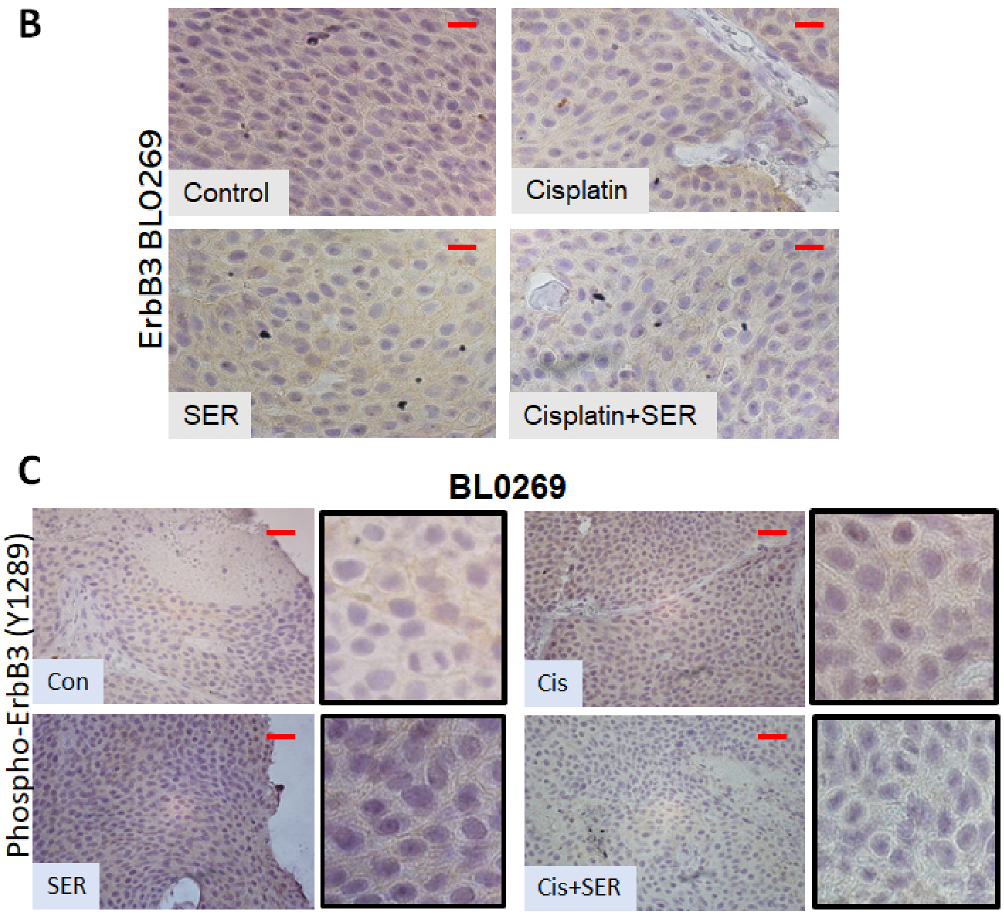

Supplementary Figure 6. Seribantumab, but not trastuzumab, reduces tumor growth in the cisplatin sensitive BL0440 PDX model and in cisplatin resistant T24 cells.
A). J82 cells were treated with vehicle, 200 nM cisplatin, 2 μM seribantumab or the combination for 72 hours, after which cells were trypsinized, and unfixed cells were stained with propidium iodide (PI) and Annexin V (AV). Stained cells were then assayed by flow cytometry. Data obtained was analyzed to obtain fractions that represent live cells (unstained), early apoptosis (Annexin V stained), late apoptosis (both AV and PI treated) and in necrosis (PI only). **B, C)** Mice dually implanted with BL0440 PDX tumors were treated with 10 mg/kg seribantumab, 10 mg/kg Herceptin (trastuzumab), or placebo twice weekly for three weeks and then monitored for up to 10 days. **B).** Corresponding box and whisker plots of tumor size. **C).** Immunohistochemical staining of ErbB3 of BL0440 PDX tumors treated with vehicle or trastuzumab. Bar: 50 μm.

A

J82

	AV-/PI-	AV+/PI-	AV+/PI+	AV-/PI+
	LIVE CELLS	EARLY APOPTOSIS	NECROTIC CELLS	LATE APOPTOSIS
Control	88.2	3.56	7.76	0.87
Cisplatin (200 nM)	***83.3***	***7.72***	***8.12***	***0.84***
Seribantumab (2 μM)	87.5	4.22	7.47	0.83
**Combo**	**69.0**	**16.8**	**13.5**	**0.7**

B

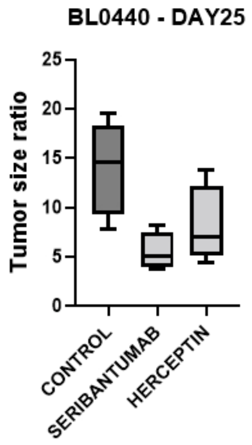

C

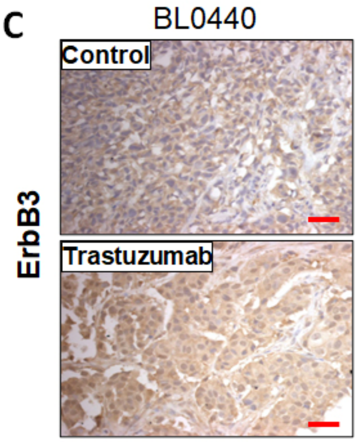

Supplement: Supplementary file 1 — Supplementary Information. [file 41598_2023_36774_MOESM1_ESM.pdf]
