# Supplementary material for: Cisplatin-induced increase in heregulin 1 and its attenuation by the monoclonal ErbB3 antibody seribantumab in bladder cancer
Source: Sci Rep. 2023 Jun 14;13:9617. doi: 10.1038/s41598-023-36774-1 (PMC10267166; doi:10.1038/s41598-023-36774-1)

Uncropped Images with Band Quantification

Western Blots

Figure 3C

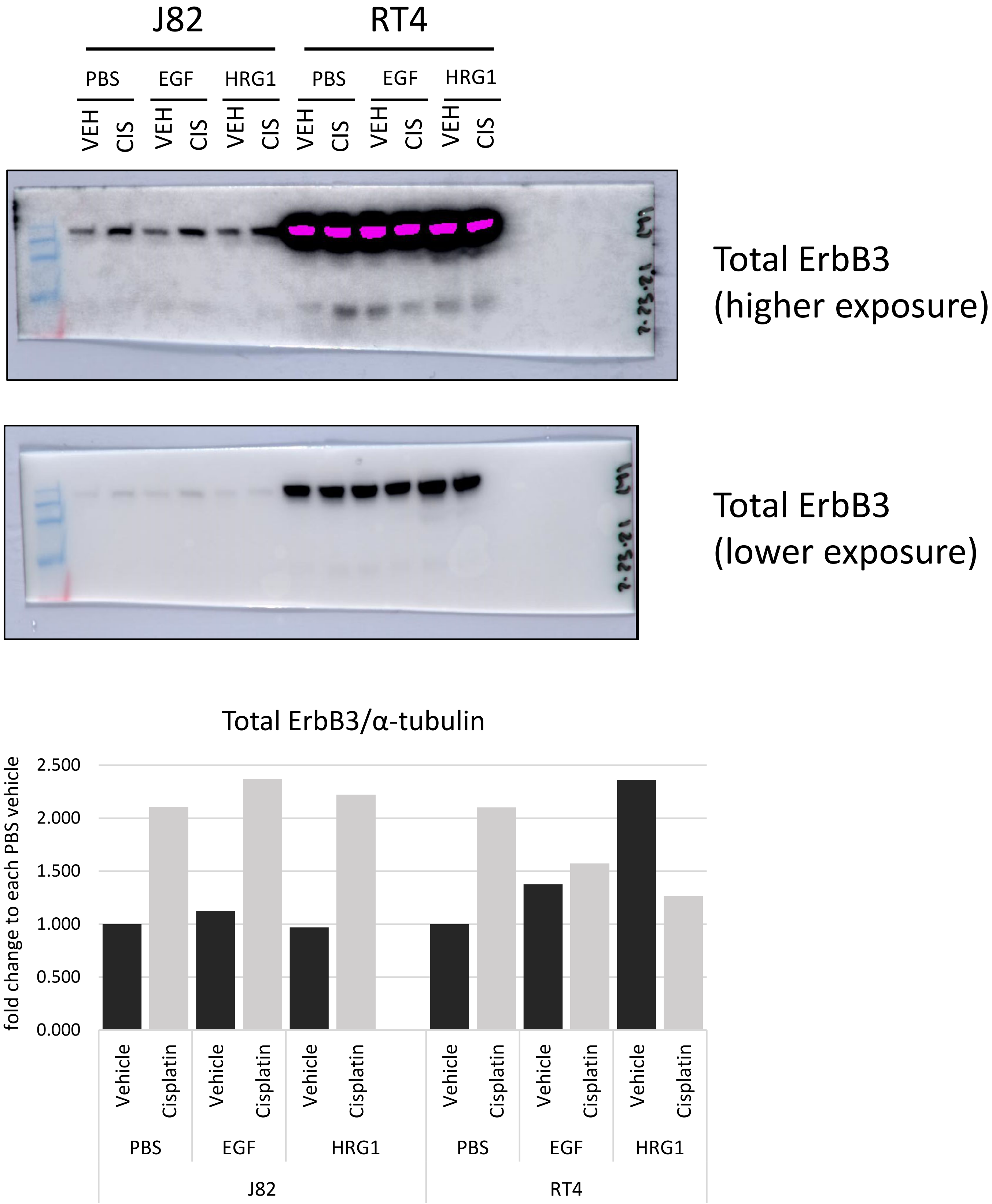

J82
RT4
PBS
EGF
HRG1
PBS
EGF
HRG1
VEH
CIS
VEH
CIS
VEH
CIS
VEH
CIS
VEH
CIS
VEH
CIS
Total ErbB3
(higher exposure)
Total ErbB3
(lower exposure)
Total ErbB3/α-tubulin
fold change to each PBS vehicle
2.500
2.000
1.500
1.000
0.500
0.000
Vehicle
Cisplatin
Vehicle
Cisplatin
Vehicle
Cisplatin
Vehicle
Cisplatin
Vehicle
Cisplatin
Vehicle
Cisplatin
PBS
EGF
HRG1
PBS
EGF
HRG1
J82
RT4

Figure 3C

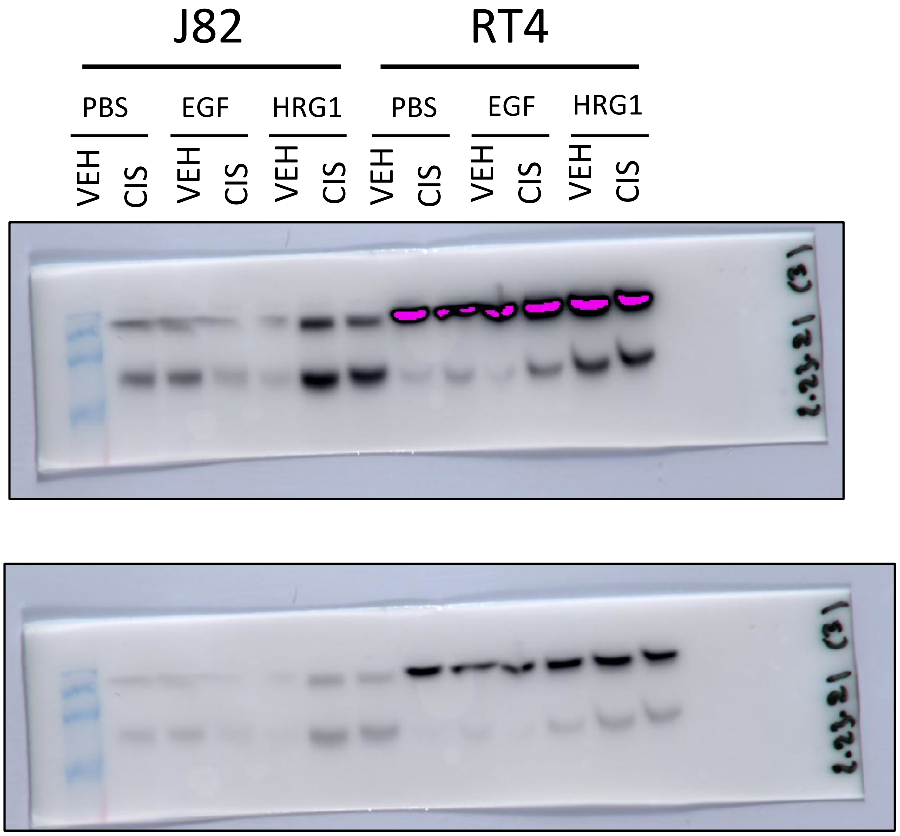

Total ErbB2
(higher exposure)

Total ErbB2
(lower exposure)

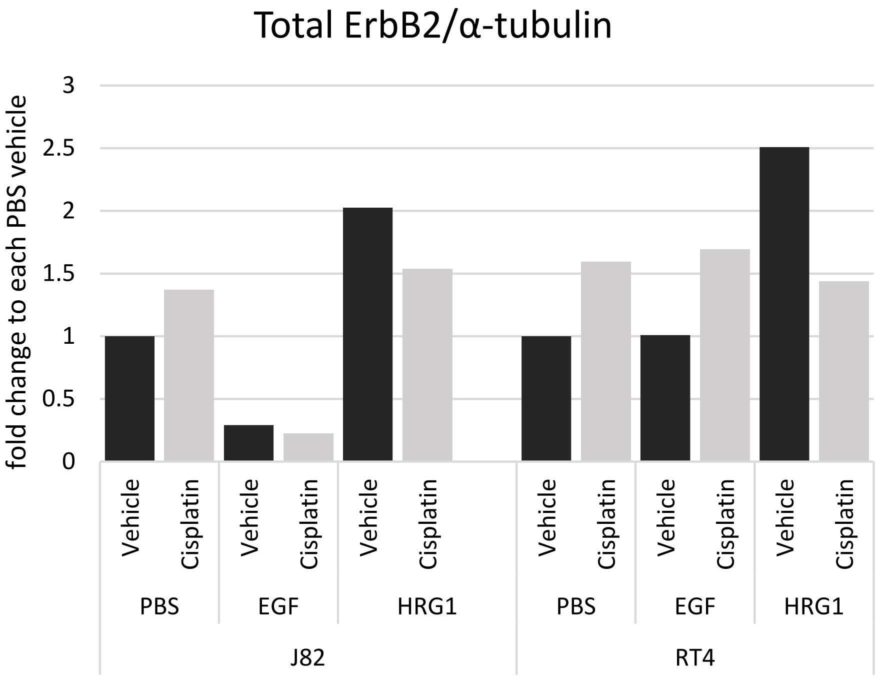

Figure 3C

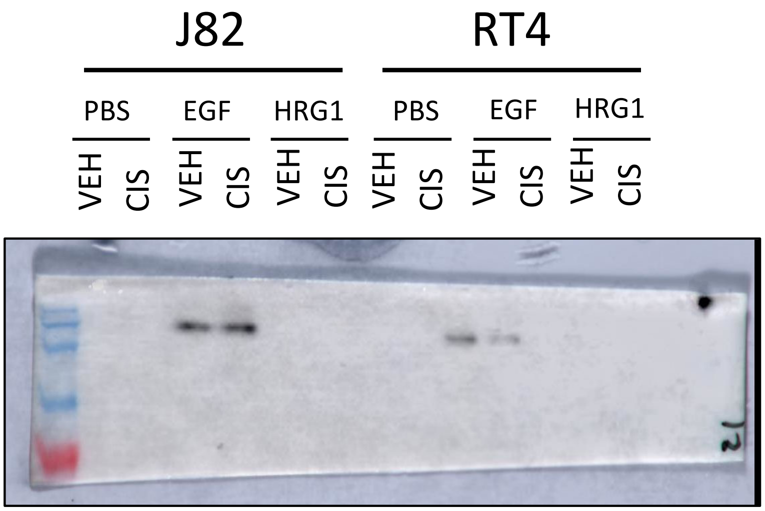

Phospho-EGFR Y1068

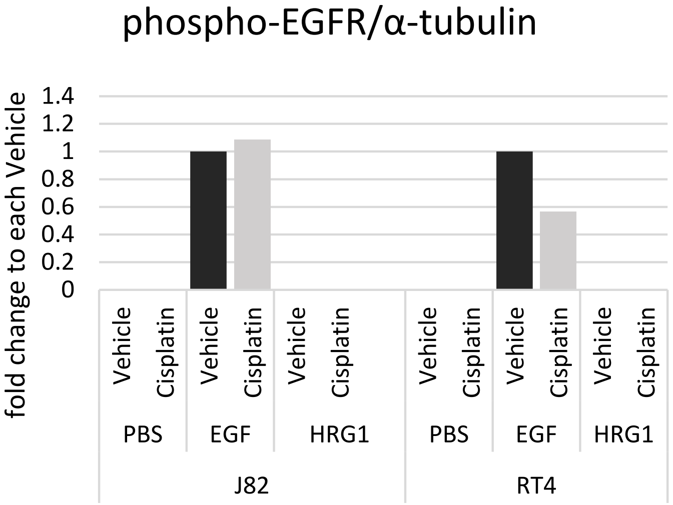

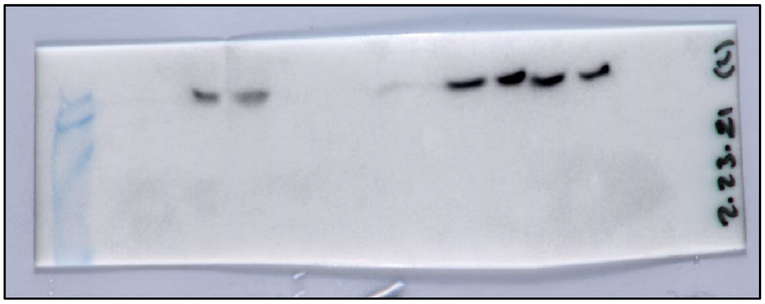

Phospho-ErbB2 Y1248

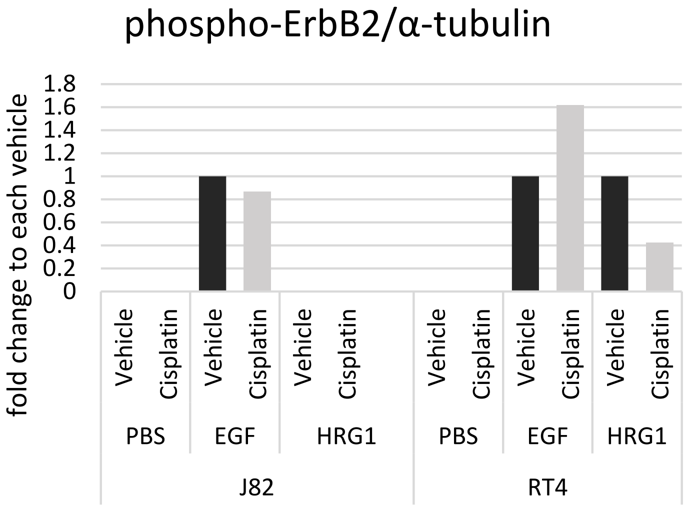

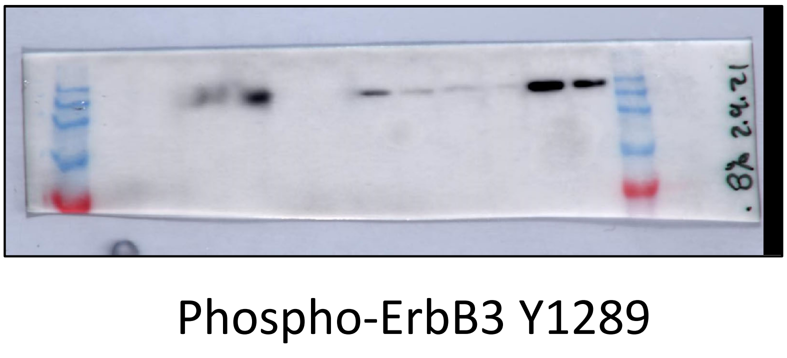

Phospho-ErbB3 Y1289

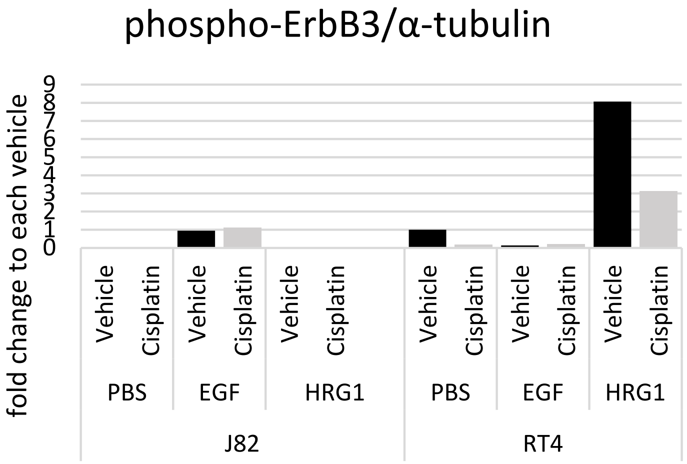

Graphs- Normalization to PBS Vehicle for J82 and RT4 individually

Figure 3C (contd)

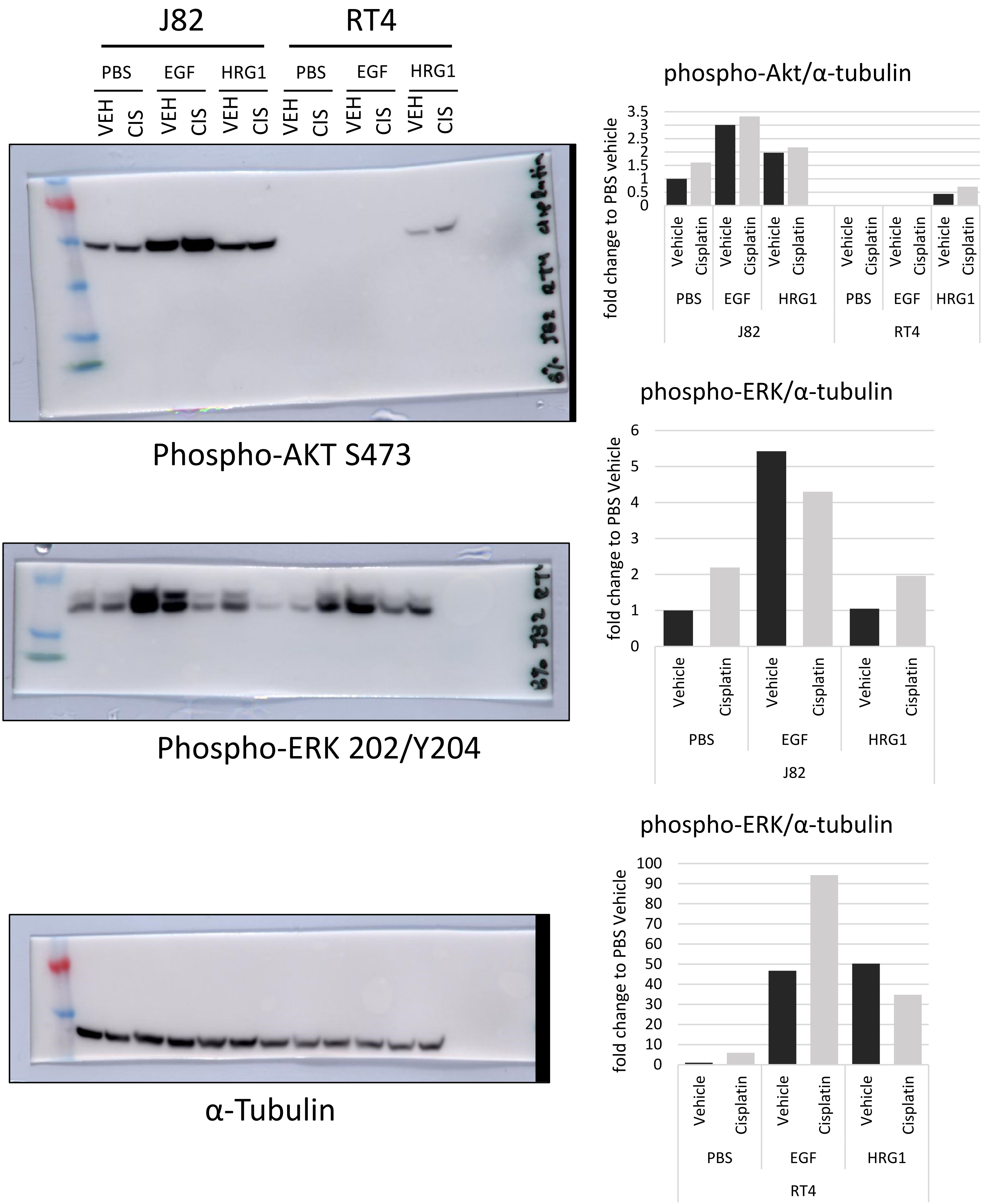

Figure 3D

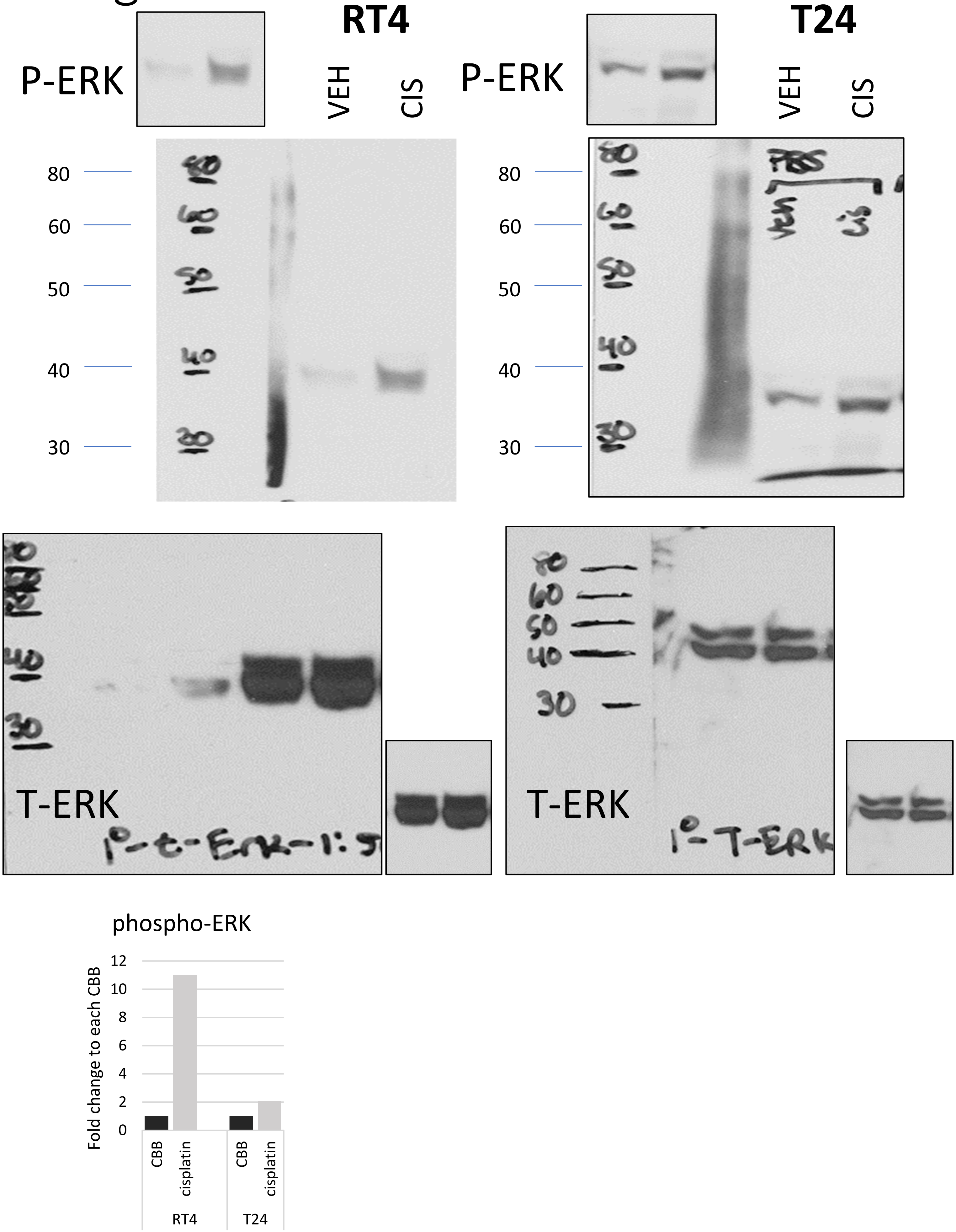
RT4
T24
P-ERK
VEH
CIS
P-ERK
VEH
CIS
80
60
50
40
30
80
60
50
40
30
T-ERK
T-ERK
phospho-ERK
Fold change to each CBB
12
10
8
6
4
2
0
CBB
cisplatin
CBB
cisplatin
RT4
T24

Figure 5A

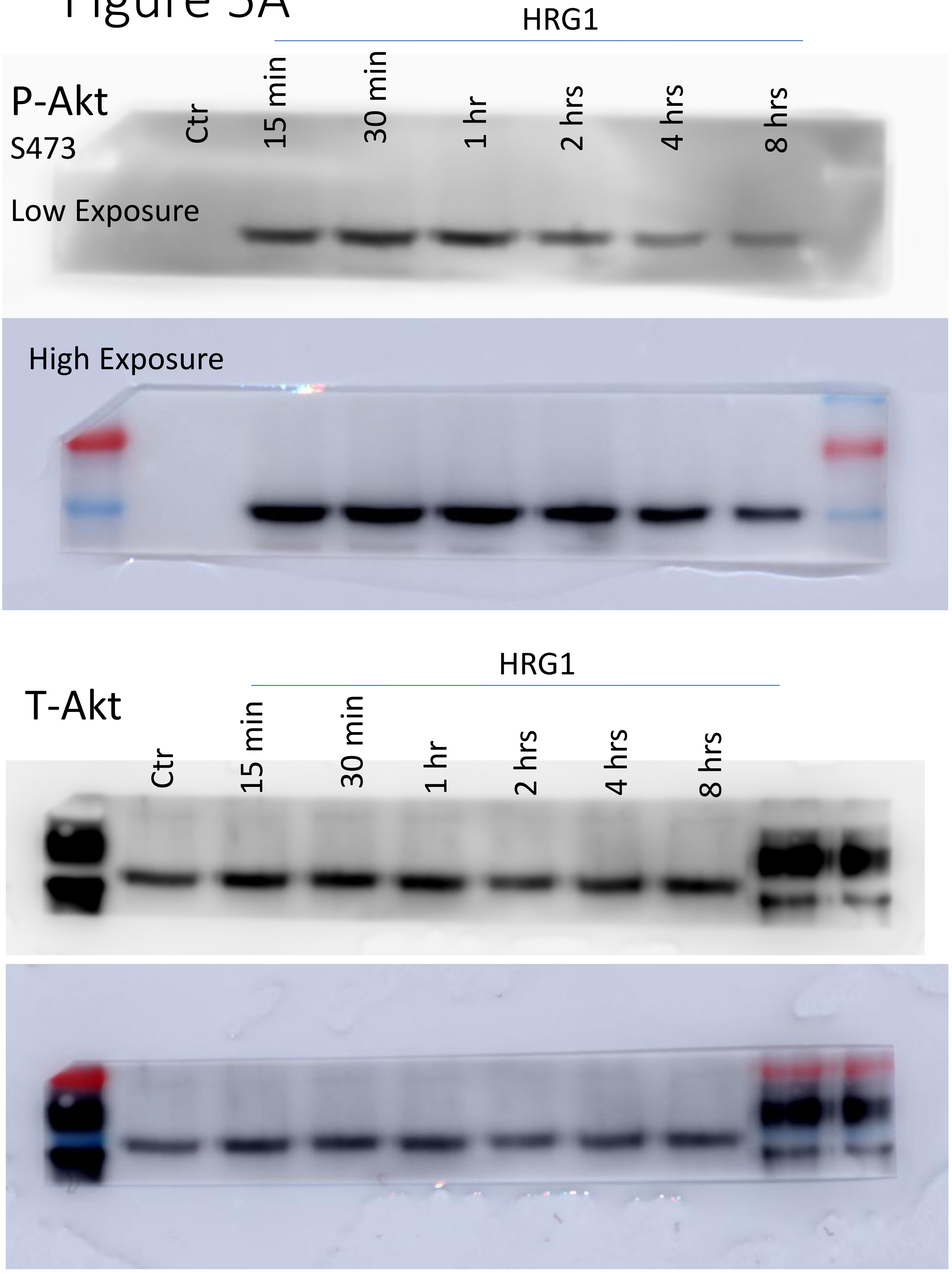
HRG1
P-Akt
S473
Ctr
15 min
30 min
1 hr
2 hrs
4 hrs
8 hrs
Low Exposure
High Exposure
HRG1
T-Akt
Ctr
15 min
30 min
1 hr
2 hrs
4 hrs
8 hrs

Figure 5A

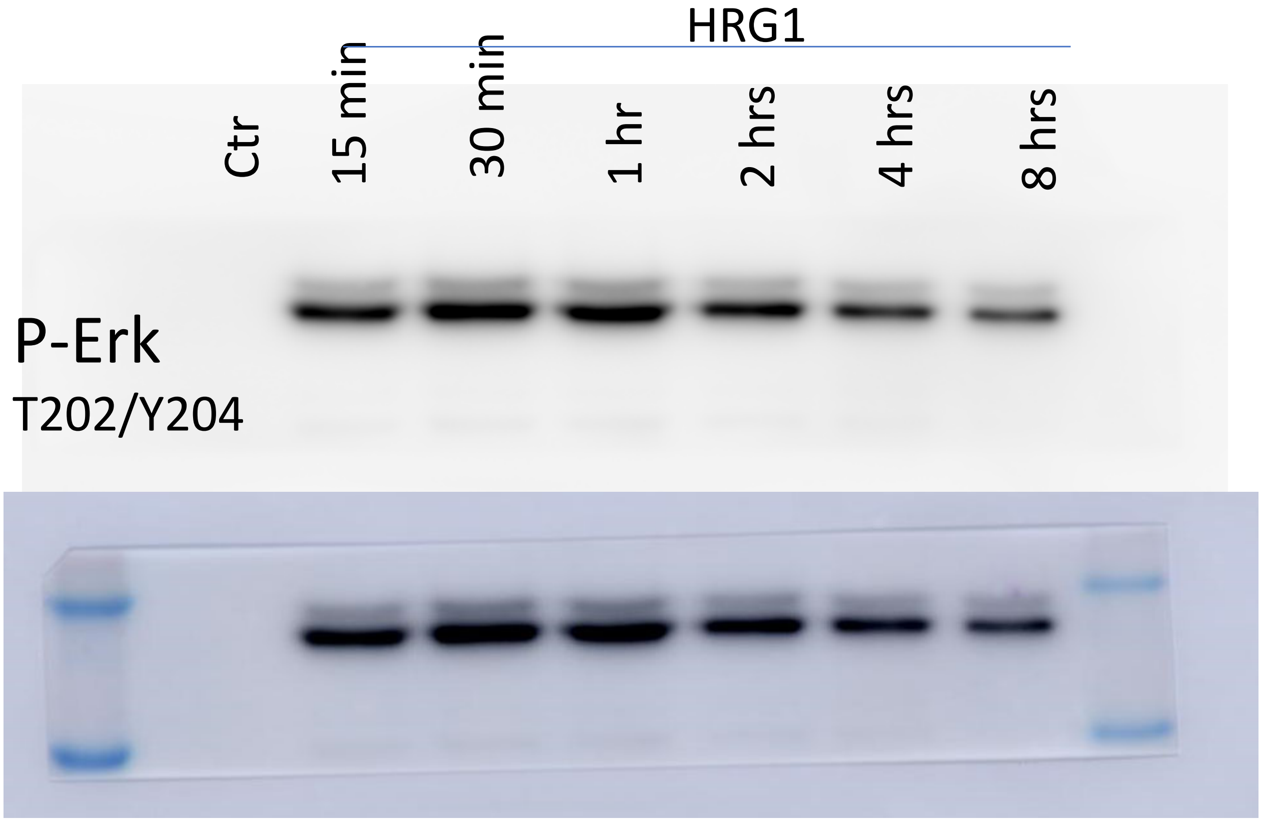

HRG1
Ctr
15 min
30 min
1 hr
2 hrs
4 hrs
8 hrs
P-Erk
T202/Y204

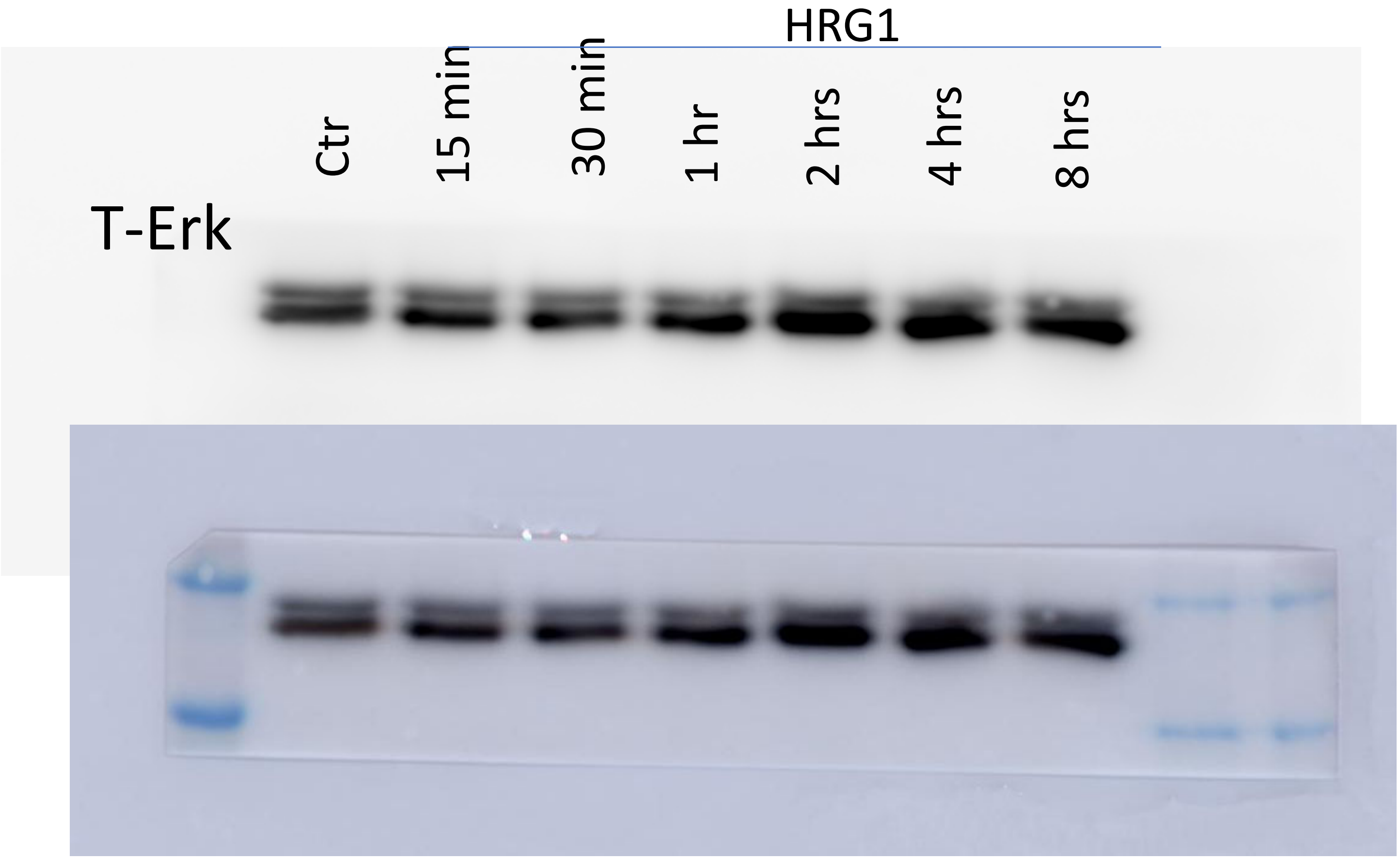

HRG1
Ctr
15 min
30 min
1 hr
2 hrs
4 hrs
8 hrs
T-Erk

Figure 5A

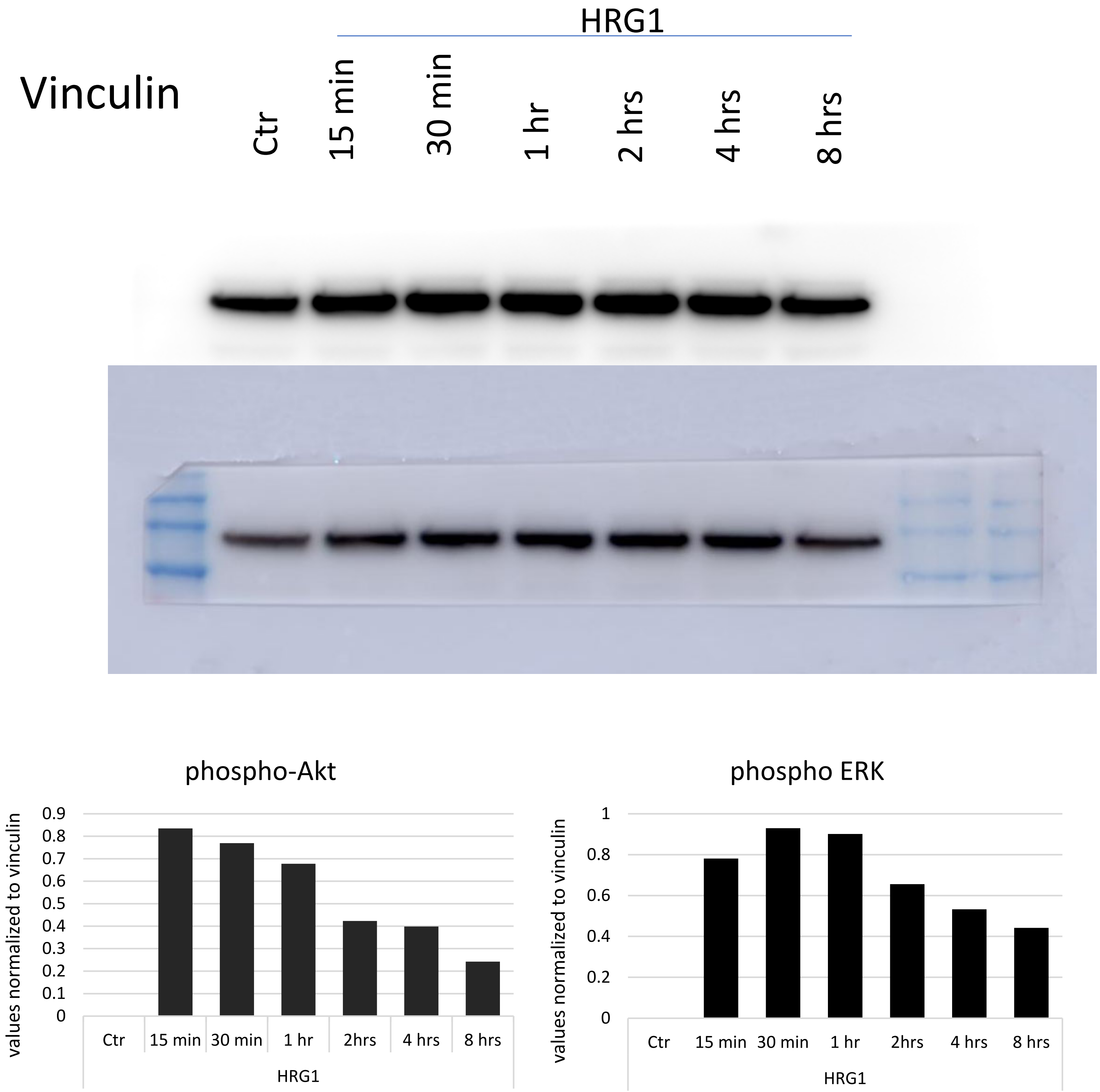

HRG1
Vinculin
Ctr
15 min
30 min
1 hr
2 hrs
4 hrs
8 hrs
phospho-Akt
values normalized to vinculin
0.9
0.8
0.7
0.6
0.5
0.4
0.3
0.2
0.1
0
Ctr
15 min
30 min
1 hr
2hrs
4 hrs
8 hrs
HRG1
phospho ERK
values normalized to vinculin
1
0.8
0.6
0.4
0.2
0
Ctr
15 min
30 min
1 hr
2hrs
4 hrs
8 hrs
HRG1

Figure 5B

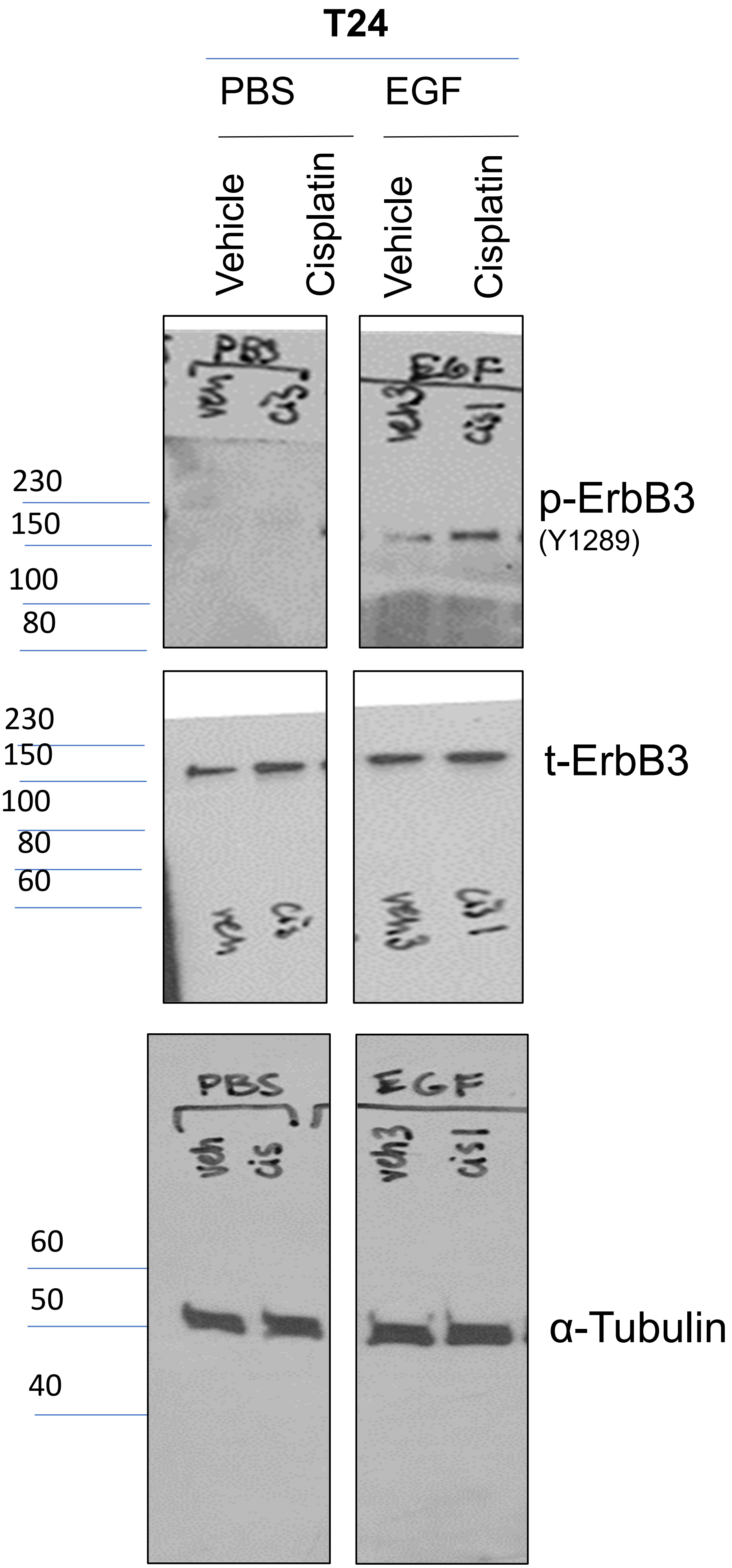
T24
PBS
EGF
Vehicle
Cisplatin
Vehicle
Cisplatin
230
150
100
80
p-ErbB3
(Y1289)
230
150
100
80
60
t-ErbB3
60
50
40
α-Tubulin

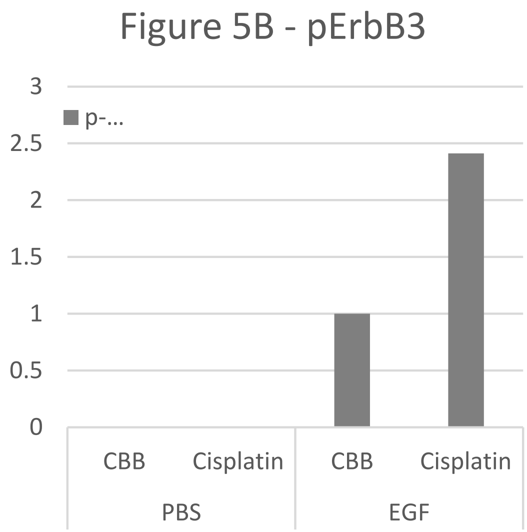
Figure 5B - pErbB3
p-...
3
2.5
2
1.5
1
0.5
0
CBB
Cisplatin
CBB
Cisplatin
PBS
EGF

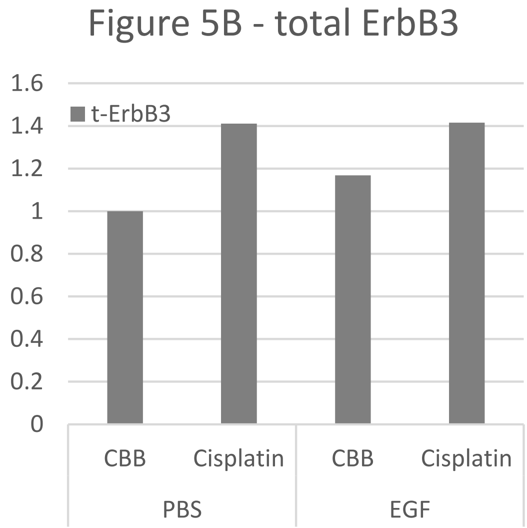
Figure 5B - total ErbB3
t-ErbB3
1.6
1.4
1.2
1
0.8
0.6
0.4
0.2
0
CBB
Cisplatin
CBB
Cisplatin
PBS
EGF

Figure 7A

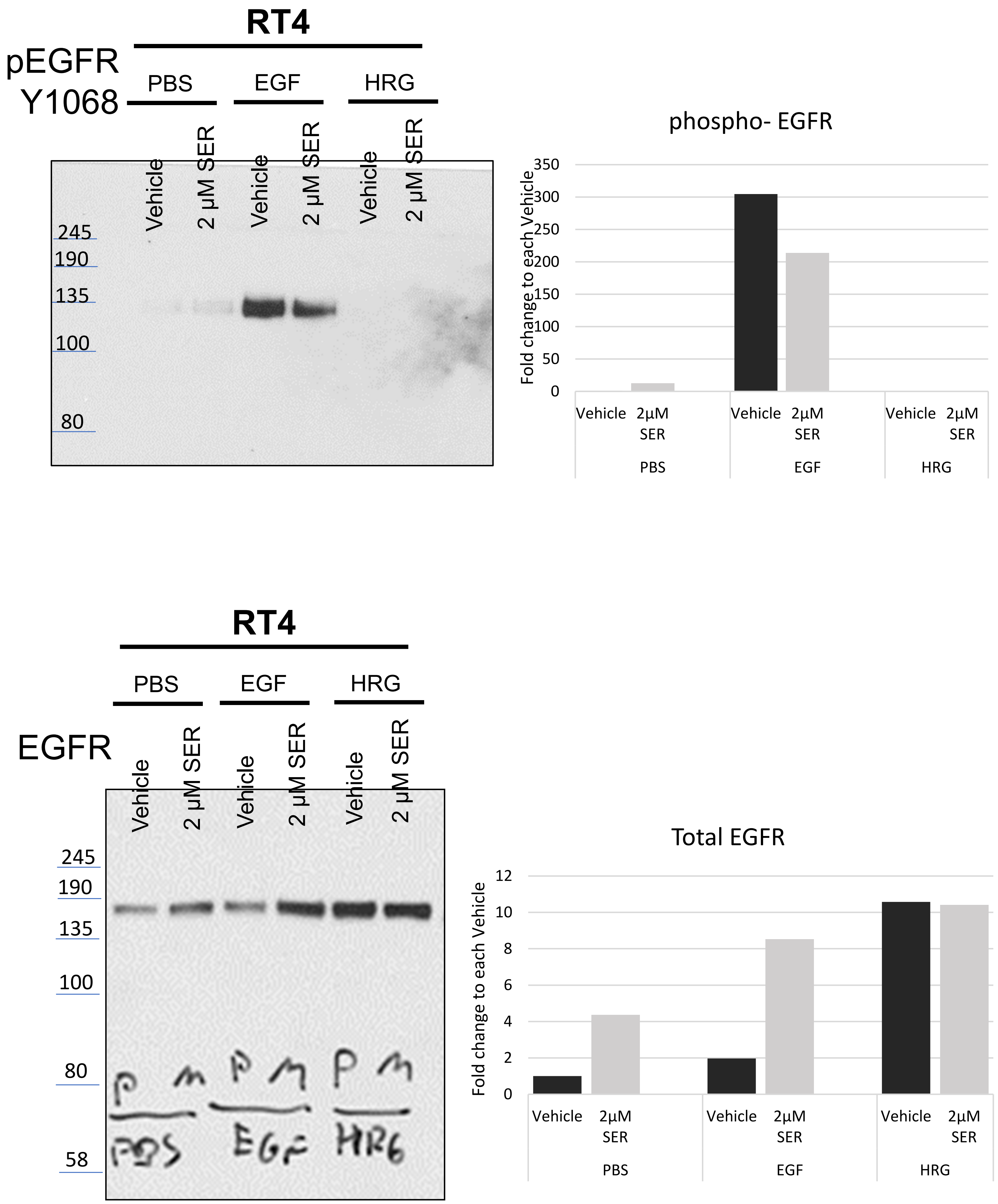

RT4
pEGFR
Y1068
PBS
EGF
HRG
Vehicle
2 μM SER
Vehicle
2 μM SER
Vehicle
2 μM SER
245
190
135
100
80
phospho- EGFR
Fold change to each Vehicle
350
300
250
200
150
100
50
0
Vehicle
2μM SER
Vehicle
2μM SER
Vehicle
2μM SER
PBS
EGF
HRG
RT4
PBS
EGF
HRG
EGFR
Vehicle
2 μM SER
Vehicle
2 μM SER
Vehicle
2 μM SER
245
190
135
100
80
58
Total EGFR
Fold change to each Vehicle
12
10
8
6
4
2
0
Vehicle
2μM SER
Vehicle
2μM SER
Vehicle
2μM SER
PBS
EGF
HRG

Figure 7A

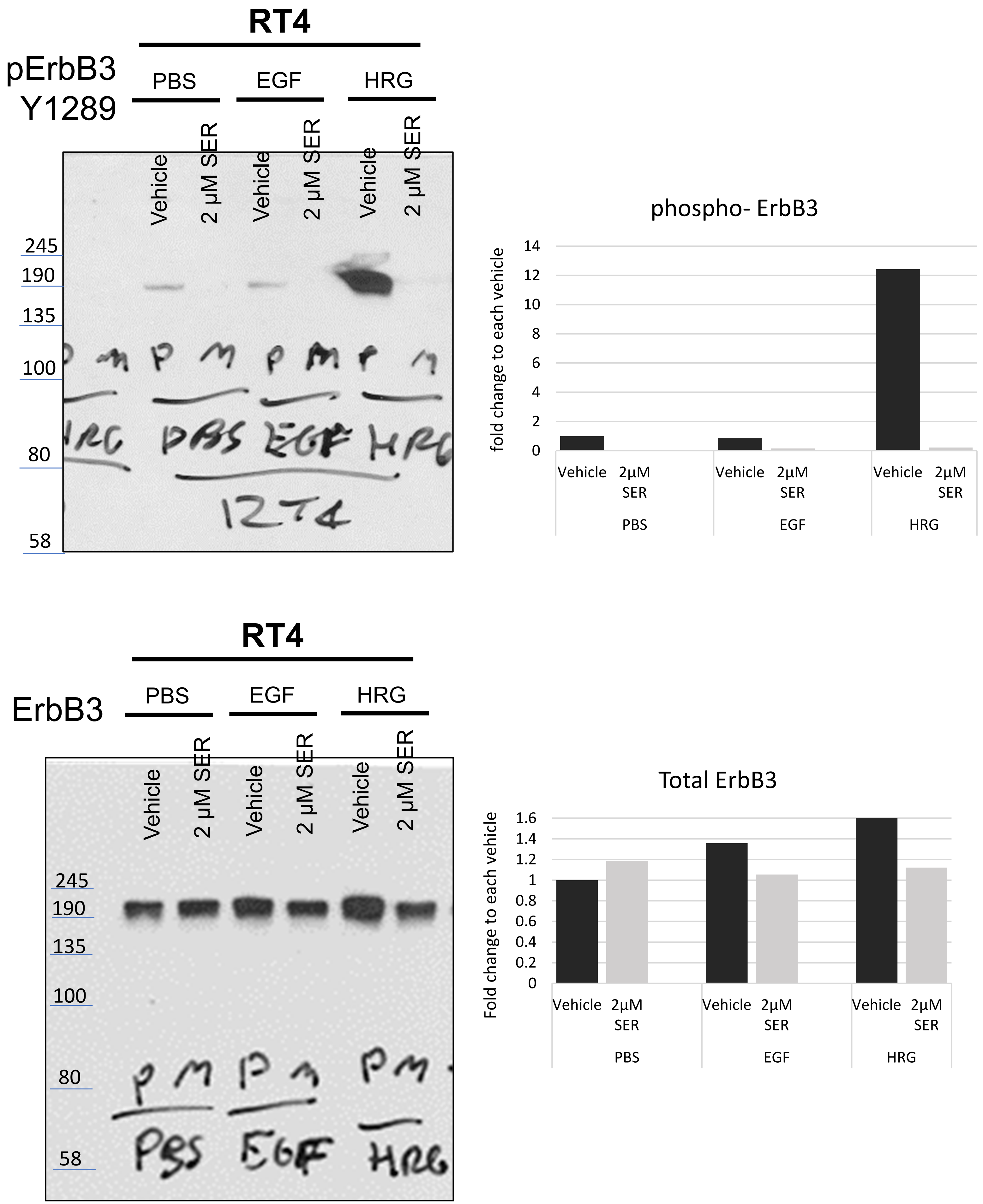
RT4
pErbB3 Y1289
PBS
EGF
HRG
Vehicle
2 µM SER
245
190
135
100
80
58
phospho- ErbB3
fold change to each vehicle
14
12
10
8
6
4
2
0
Vehicle
2µM SER
PBS
EGF
HRG
RT4
ErbB3
PBS
EGF
HRG
Vehicle
2 µM SER
245
190
135
100
80
58
Total ErbB3
Fold change to each vehicle
1.6
1.4
1.2
1
0.8
0.6
0.4
0.2
0
Vehicle
2µM SER
PBS
EGF
HRG

Figure 7A

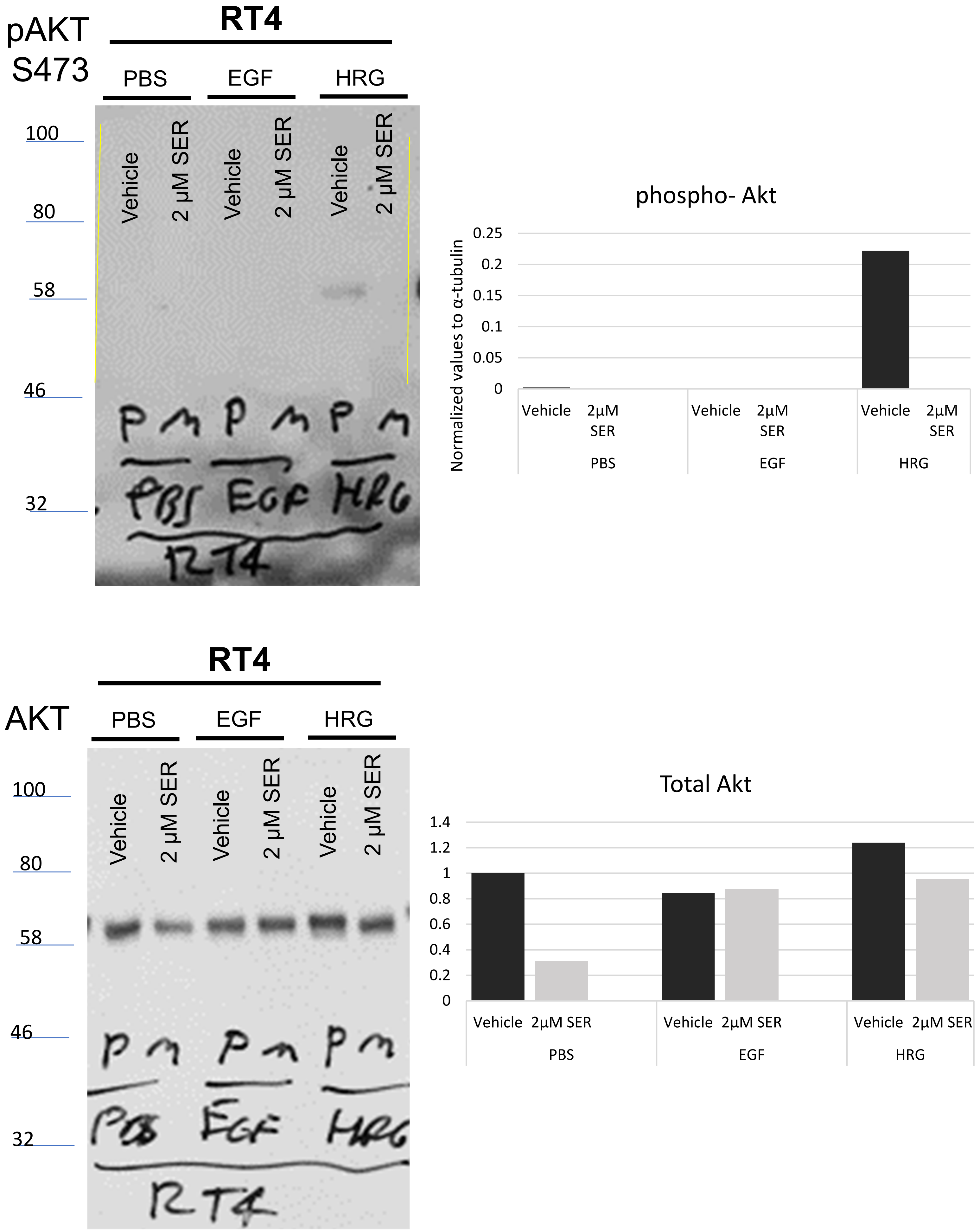
pAKT S473
RT4
PBS
EGF
HRG
Vehicle
2 µM SER
100
80
58
46
32
phospho- Akt
Normalized values to α-tubulin
0.25
0.2
0.15
0.1
0.05
0
Vehicle
2µM SER
PBS
EGF
HRG
AKT
RT4
Total Akt
1.4
1.2
1
0.8
0.6
0.4
0.2
0

Figure 7A

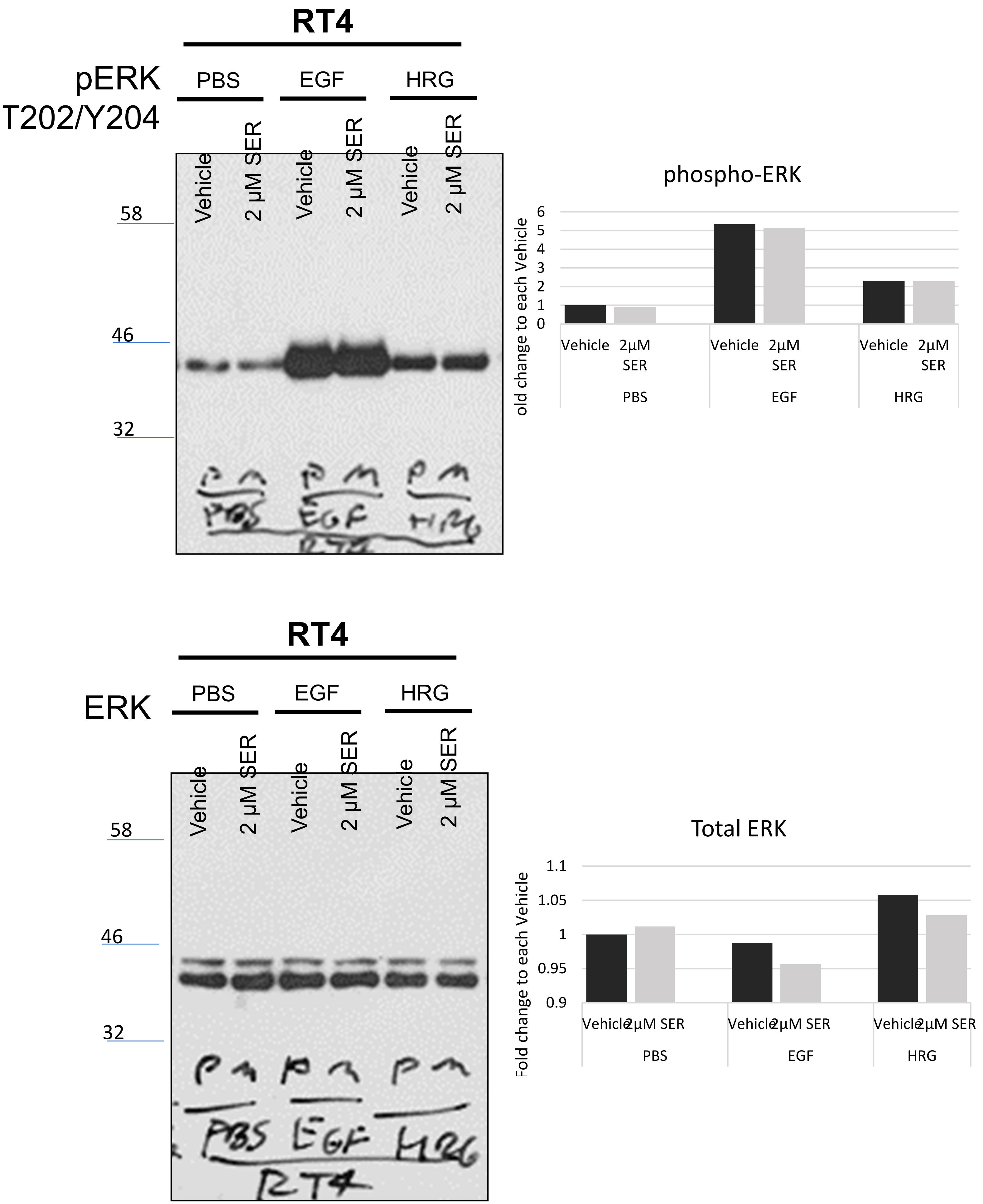
RT4
pERK
T202/Y204
PBS
EGF
HRG
Vehicle
2 µM SER
58
46
32
phospho-ERK
Fold change to each Vehicle
6
5
4
3
2
1
0
Vehicle
2µM SER
PBS
EGF
HRG
RT4
ERK
PBS
EGF
HRG
Vehicle
2 µM SER
58
46
32
Total ERK
Fold change to each Vehicle
1.1
1.05
1
0.95
0.9
Vehicle
2µM SER
PBS
EGF
HRG

Figure 7A

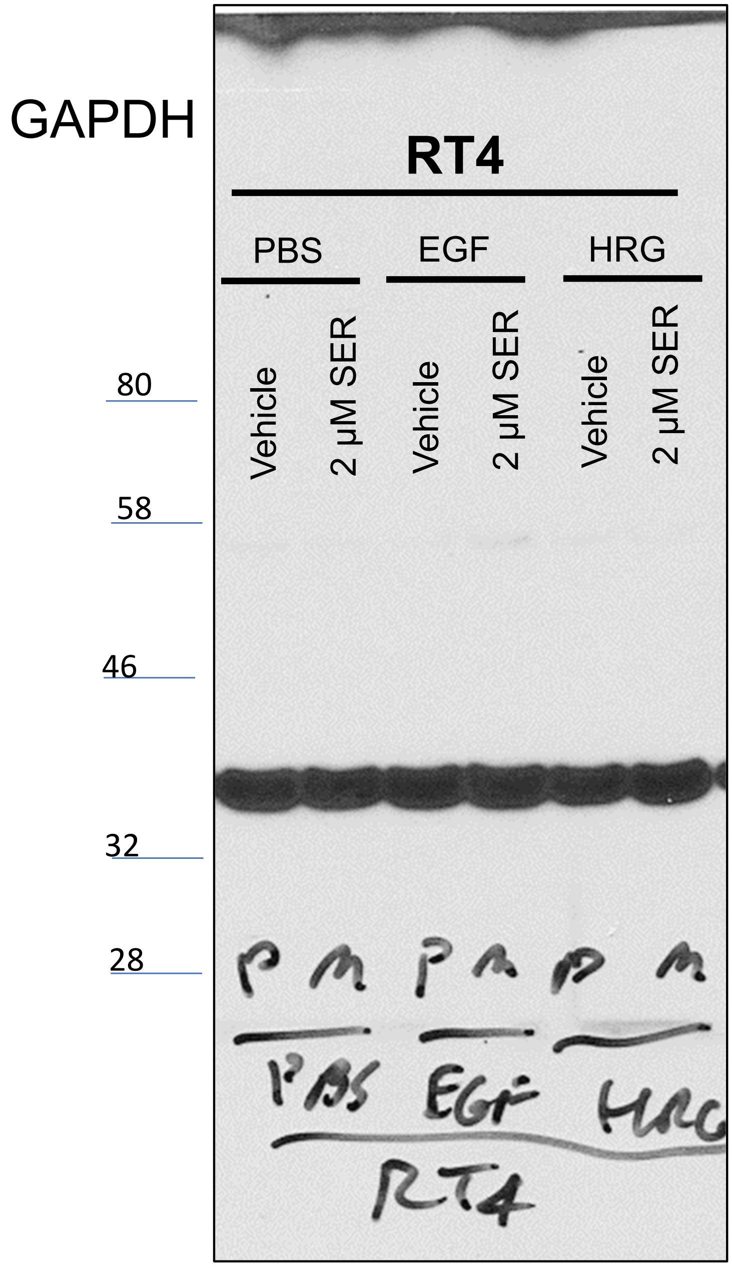
GAPDH
RT4
PBS
EGF
HRG
Vehicle
2 µM SER
Vehicle
2 µM SER
Vehicle
2 µM SER
80
58
46
32
28

Supplementary Figure 3A

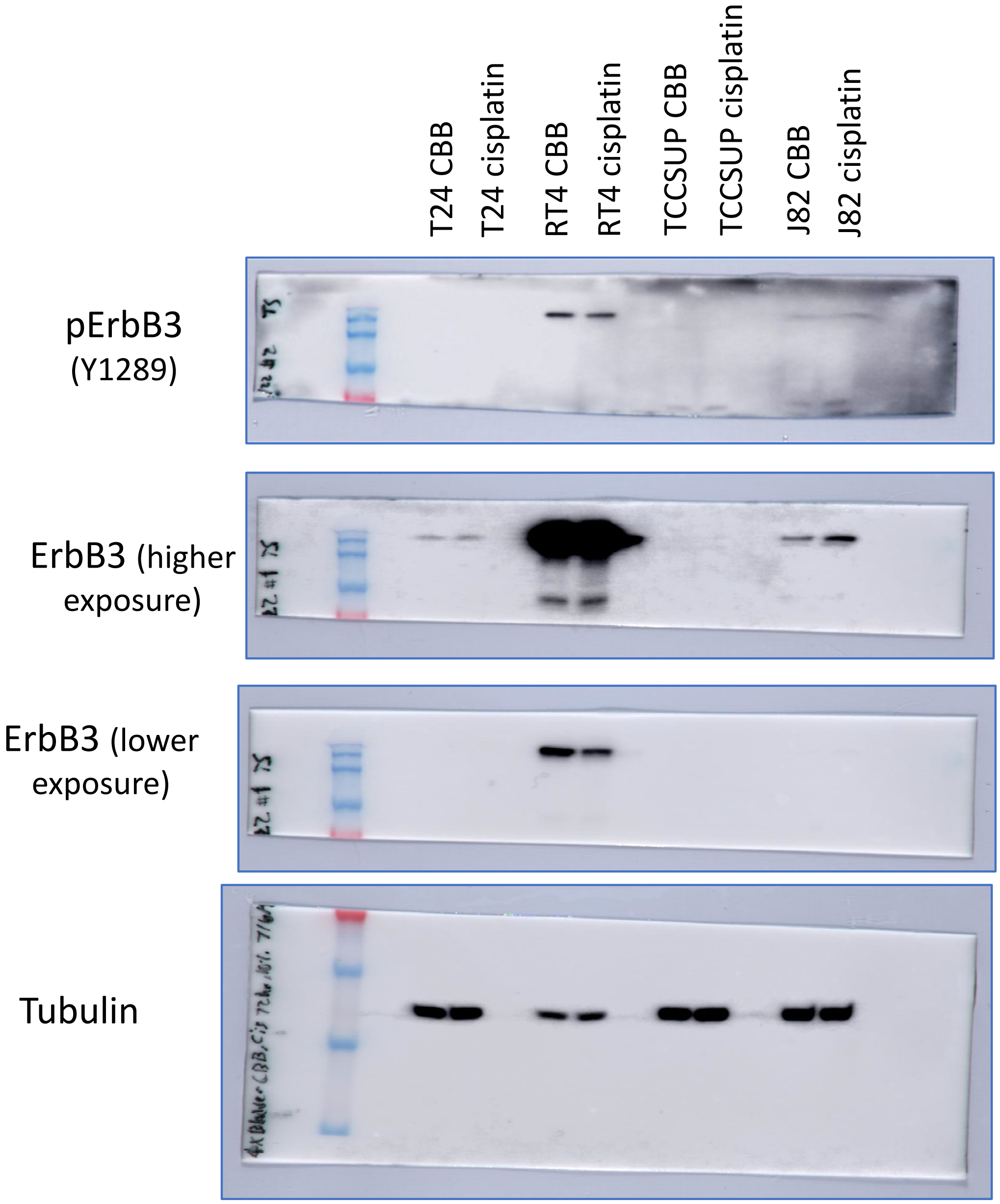

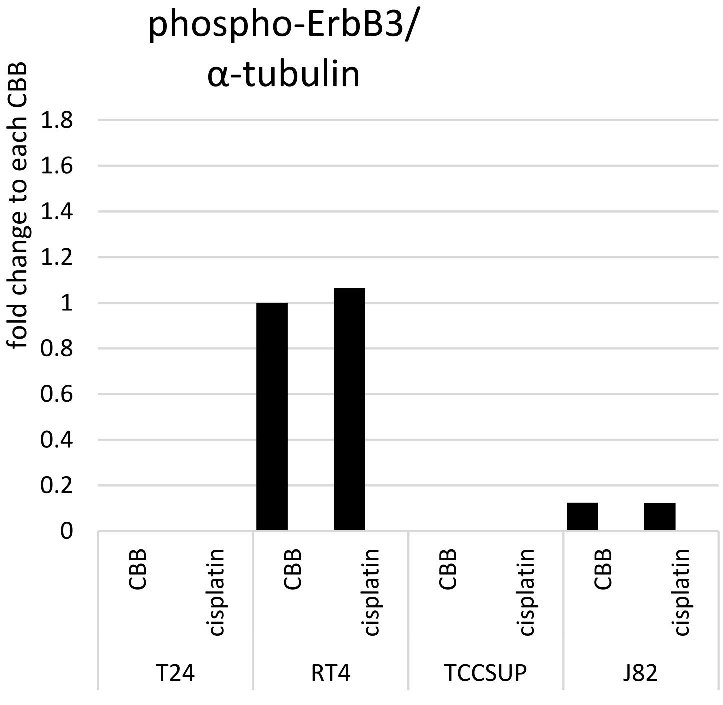

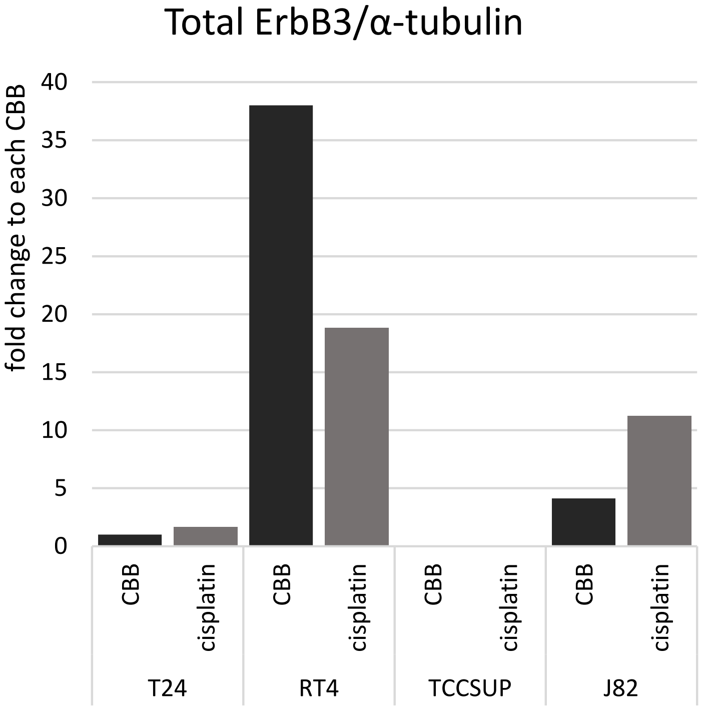

Supplement: Supplementary file 2 — Supplementary Figures. [file 41598_2023_36774_MOESM2_ESM.pdf]
